# Impact of *Kappaphycus alvarezii* Biostimulant on Growth, Biochemistry, Essential Oil, and Rhizosphere of Basil (*Ocimum basilicum*) Plants

**DOI:** 10.3390/plants15111749

**Published:** 2026-06-04

**Authors:** Aline Nunes, Luana Vanessa Peretti Minello, Eva Regina Oliveira, Alex Ricardo Schneider, Felipe de Souza Dutra, Tainara Guizolfi, Lohan Rodrigues Brandão Santos, Valéria Cress Gelli, Camille Eichelberger Granada, Raul Antonio Sperotto, Sidnei Moura, Marcelo Maraschin, Giuseppina Pace Pereira Lima

**Affiliations:** 1 Plant Biotechnology and Postharvest Laboratory, Department of Chemical and Biological Sciences, Institute of Biosciences, São Paulo State University, Botucatu 18618-970, Brazil; pace.lima@unesp.br; 2 Graduate Program in Plant Physiology, Botany Department, Biology Institute, Federal University of Pelotas, Pelotas 96010-610, Brazil; lvpminello@gmail.com (L.V.P.M.); raul.sperotto@embrapa.br (R.A.S.); 3 Laboratory of Metabolomics and Applied Biochemistry, Department of Plant Science, Federal University of Santa Catarina, Florianópolis 88034-000, Brazil; ginagro@gmail.com (E.R.O.); arschneider1@ucs.br (A.R.S.); felipedutra.rez@gmail.com (F.d.S.D.); lohanbrandao645@gmail.com (L.R.B.S.); m2@cca.ufsc.br (M.M.); 4 Laboratory of Biotechnology of Natural and Synthetic Products, Institute of Biotechnology, University of Caxias do Sul, Caxias do Sul 95070-560, Brazil; tguizolfi@ucs.br (T.G.); smsilva11@ucs.br (S.M.); 5 Fisheries Institute, APTA at Secretariat of Agriculture and Supplies of São Paulo State, São Paulo 11680-000, Brazil; valeria.gelli@sp.gov.br; 6 Graduate Program in Genetics and Molecular Biology, Biosciences Institute, Genetics Department, Federal University of Rio Grande do Sul, Porto Alegre 91501-970, Brazil; camilleegranada@gmail.com; 7 Brazilian Agricultural Research Corporation (Embrapa Maize and Sorghum), Sete Lagoas 35702-098, Brazil

**Keywords:** biofertilizer, rhizosphere, Rhodophyta

## Abstract

Seaweed-derived biostimulants are a promising strategy for improving crop performance in sustainable agriculture. In this context, this study evaluated the effects of foliar application of *Kappaphycus alvarezii* extracts, obtained from two Brazilian regions (São Paulo: Kal-SP and Santa Catarina: Kal-SC), at different concentrations (1%, 3%, 5%, and 7%) on the growth, biochemical profile, essential oil yield, and rhizosphere microbiome of *Ocimum basilicum* under field conditions. Morphological analysis indicated that the 5% and 7% concentrations increased plant height, biomass, root development, and inflorescence production, with biomass gains of up to 51% and essential oil production increases of up to 142% compared to the control. Biochemical responses varied by extract origin, with Kal-SC promoting greater increases in photosynthetic pigments, antioxidant activity, and carbon-related metabolites, whereas Kal-SP induced only minor metabolic changes. The algal biostimulant modulated essential oil yield and composition, promoting treatment-dependent shifts in major terpenoid compounds. Microbiome analysis showed no significant changes in alpha diversity, but significant shifts in beta diversity and functional groups, such as *Bacillaceae*, indicating rhizosphere reorganization. Overall, the effectiveness of *K. alvarezii*-based biostimulants depends on concentration and biomass source, highlighting their potential as sustainable agricultural bioproducts and the importance of standardized extraction for consistent outcomes.

## 1. Introduction

In recent years, the pursuit of more sustainable agricultural practices has driven the adoption of various approaches to promote plant growth and health, particularly in response to the extensive impacts of climate change on the agricultural sector [1,2]. Plant inoculation with natural products contributes to the development of sustainable practices, as their use can enhance nutrient absorption efficiency, improve resistance to environmental stressors, and stimulate essential physiological processes [3,4].

The use and application of biostimulants have increased in recent years, with a report from Fortune Business Insights estimating that the global biostimulant market was valued at USD 4.54 billion in 2025 and is projected to reach USD 12.86 billion by 2034, representing a compound annual growth rate (CAGR) of 12.34% [5]. Biostimulants can be categorized primarily based on their main component or mode of action. They can be of biological origin, with components and molecules derived from plants or other organic sources, or of physical or chemical origin [4].

Among these sources, biostimulants derived from seaweed extracts stand out due to their rich composition of bioactive compounds, including amino acids, polysaccharides, vitamins, fatty acids, minerals, phenolics, and phytohormones [6]. In this context, the red alga *Kappaphycus alvarezii* (Rhodophyta) has been investigated as a biostimulant across various crop species. A systematic review of research conducted between 2017 and 2023 identified 34 manuscripts focused on their agricultural applications. Notable benefits include enhanced plant quality and productivity, improved physical attributes and quality of grains, increases in biochemical traits and micronutrients, regulation of genes responsible for plant growth and development, reduced greenhouse gas emissions, improved germination rates, extended storage times, greater fruit production, induction of rooting, and increased resistance to drought and salinity stress, and pathogen infection [7]. In addition, seaweed extracts may modulate the soil microbiota, potentially enhancing the α-diversity and functional activity of rhizosphere bacteria [8,9]. This effect occurs through biostimulant-soil–plant interaction, leading to positive impacts on agronomic parameters [10].

However, despite the numerous successful findings reported, several factors can influence the nutritional and biochemical composition of *K. alvarezii*, directly affecting its effectiveness as a biostimulant. Environmental conditions at the cultivation site, such as temperature and salinity, can promote significant compositional changes in the macroalga [11,12,13]. Nevertheless, the influence of these environmental variations on the composition of *K. alvarezii* extracts and their subsequent agricultural applications remains poorly understood. In addition, the effects of biostimulant concentration used in agricultural practices should also be considered [14].

In this context, *Ocimum basilicum* is an important model species due to its high economic and medicinal value, particularly for producing essential oils widely used in the food, pharmaceutical, and cosmetic industries. Moreover, the yield and chemical composition of basil essential oil are strongly influenced by environmental and cultivation conditions [15,16], making this species particularly suitable for evaluating the effects of seaweed-derived biostimulants on plant growth, metabolism, and rhizosphere interactions under field conditions. Thus, this study aimed to clarify the synergistic regulation mechanisms of *K. alvarezii* extracts produced in two Brazilian states (São Paulo/Southeast Region and Santa Catarina/South Region) at different concentrations on *O. basilicum* morphogenesis, secondary metabolism, and rhizosphere microecology under field conditions.

## 2. Results

### 2.1. Environmental Data

The analyzed environmental data indicated that precipitation varied from 0 to 146.60 mm (Figure S2, Supplementary Material). A total of 42 days recorded no rainfall, while 14 days experienced low precipitation (0.20 to 2 mm), 15 days had moderate rain (2 to 9 mm), and 10 days recorded high rainfall (11 to 146.60 mm).

Regarding relative humidity, values ranged from 69.92% to 96.41%, indicating consistently high humidity throughout the experimental period (Figure S2, Supplementary Material). Air temperature ranged from 20.1 °C (April 2025) to 28.6 °C (February 2025). Overall, the highest averages were recorded in February, while the lowest occurred in April, as expected for a humid subtropical climate (Figure S2, Supplementary Material).

### 2.2. Morphological Parameters

The inflorescence count was conducted three times at 15-day intervals. Statistically significant differences were observed across all time points (Table 1). In the first count, the plants treated with the biostimulant produced in São Paulo state (Kal-SP) showed a notable 5% increase, which differed significantly from all other treatments and represented a 666% increase compared to the control. For the first count involving the plants treated with the bioproduct from Santa Catarina state (Kal-SC), the treatments at 3%, 5%, and 7% did not differ from each other, but were significantly different from the others, showing increases of 245%, 654%, and 465%, respectively, with respect to the control.

In the second time-point assessment, the 5% and 7% treatments of both Kal-SP and Kal-SC biostimulants stood out significantly from the others. For Kal-SP, increases by 255% (5%) and 171% (7%) were detected as compared to the control. Similarly, the Kal-SC bioinput increased the number of flowers by 208% and 261% at concentrations of 5% and 7%, respectively. In the final count, these same treatments continued to show prominence, with Kal-SP at 5% and 7% exhibiting increases of 157% and 124%, respectively, compared to the control, and Kal-SC at 5% and 7% showing increases of 126% and 208%, respectively (Table 1).

At the end of the experiment, morphological analysis revealed statistically significant differences overall, except for the number of branches (NB) in the Kal-SP treatment, and for root dry mass (RDM) and shoot dry mass (SDM) in the Kal-SC treatment (Table 2). Plant height (PH) showed a notable response in the Kal-SP 5% treatment, followed by the 7% treatment, both of which differed significantly from each other and from the other treatments, with increases of 14.89% and 9.81%, respectively, compared to the control. Kal-SC-treated plants at 3% and 5% concentrations stood out, followed by the 1% and 7% concentrations, all differing from the control with increments of 17.34%, 17.93%, 9.05%, and 14.33%, respectively (Table 2).

Regarding the root length (RL) for the Kal-SP treatment, the highest averages were recorded for the control, 5%, and 7% treated plants, which did not differ from each other, but were significantly different from the other treatments. Similarly, for Kal-SC, the highest averages were found in the control and 7% treatments, again not differing from each other, but differing from the others (Table 2).

For the number of branches (NB) in the Kal-SC treatment, the highest averages were observed in the control, 1%, and 7% treatments, which did not differ statistically from each other and were significantly different from the other treatments. However, for the number of nodes (NN), increases of 9.29% and 10.98% were observed in Kal-SC-treated plants at 3% and 7%, respectively, compared to the control. In the Kal-SP treatment, increments of 9.29% and 11.02% were recorded at 1% and 3% concentrations, respectively (Table 2).

The root fresh mass (RFM) of the Kal-SP 5% and 7% treated plants increased by 36.73% and 35.00%, respectively, compared to the control, and differed significantly from the other treatments. Similarly, the 7% Kal-SC treatment differed statistically from the other treatments, showing a 17.61% increase over the control. For shoot fresh mass (SFM), the Kal-SP 3%, 5%, and 7% treatments did not differ statistically from each other but were significantly higher than the control, with increases of 29.88%, 51.19%, and 49.31%, respectively. In the Kal-SC-treated plants, all biostimulant concentrations (1%, 3%, 5%, and 7%) differed from the control, with increases of 40.04%, 46.99%, 51.43%, and 44.77%, respectively (Table 2).

Regarding root dry mass (RDM) and shoot dry mass (SDM), the Kal-SP 5% and 7% treatments differed significantly from the other treatments, with increments of 32.65% and 32.06% for RDM and 56.73% and 58.74% for SDM, respectively, compared to the control (Table 2).

**Figure 1:**
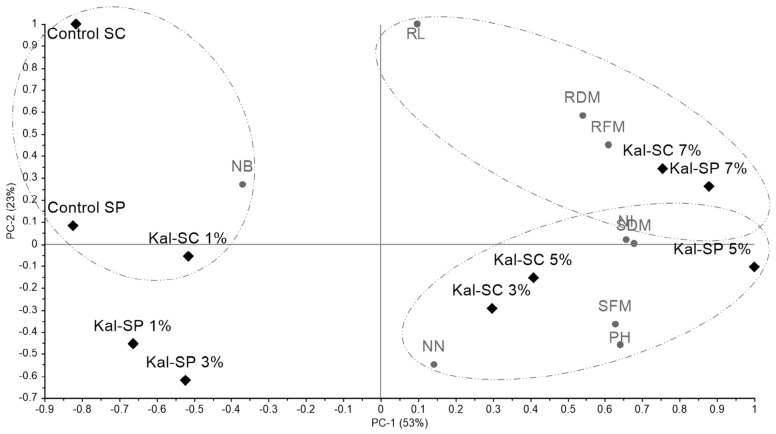
Principal component analysis (PC1 and PC2) calculated from the dataset of number of inflorescences (NI), plant height (PH), root length (RL), number of branches (NB), number of nodes (NN), root fresh mass (RFM), shoot fresh mass (SFM), root dry mass (RDM), and shoot dry mass (SDM) of basil plants treated with foliar application of *Kappaphycus alvarezii* biostimulant (1%, 3%, 5%, and 7%) cultivated in the States of São Paulo (Kal-SP) and Santa Catarina (Kal-SC), Brazil.

Upon conducting a principal component analysis (PCA) of the morphological dataset, which included the findings from the latest analysis of inflorescence number in Kal-SC- and Kal-SP-treated plants, a total variance of 76% was captured by the descriptive model, with PC1 accounting for 53% of the dataset variation (Figure 1; Table S2, Supplementary Material). Three groupings were observed: the control SP, control SC, and Kal-SC 1% were clustered according to their NB; Kal-SP and Kal-SC 7% were clustered due to their RL, RFM, RDM, NI, and SDM. Finally, Kal-SP 5%, Kal-SC 3%, and Kal-SC 5% were clustered with PH, NN, and SFM.

### 2.3. Biochemical Parameters

Statistically significant differences were observed for chlorophyll *a* (Chl *a*), chlorophyll *b* (Chl *b*), and total chlorophyll (TChl) only in the Kal-SC-treated plants, as well as for carotenoids (TCN) and antioxidant activity (DPPH assay). For total flavonoid content (TFC), significant differences were found only for Kal-SP, while total phenolic content (TPC) showed statistical differences for both Kal-SP and Kal-SC (Table 3).

For Chl *a*, Kal-SC 3%, 5%, and 7% differed statistically from the other treatments, with increments of 15.00%, 27.50%, and 17.50%, respectively, compared to the control. In terms of Chl *b*, these same treatments also exhibited higher levels than the control and Kal-SC 1%, although Kal-SC 5% differed statistically from Kal-SC 3% and 7%. Compared with control plants, Kal-SC 5% increased pigment content by 28.57%. Similar results were observed for TChl, with a 25.93% increase for Kal-SC, representing a 5% increase relative to the control. For TCN, Kal-SC 3%, 5%, and 7% differed significantly from the other treatments, with increases of 14.46%, 19.28%, and 15.66%, respectively (Table 3).

Higher TPCs were found in both control and Kal-SP-treated plants at 3%. For Kal-SC, higher TPC levels were observed in the 1% and 5% treatments, representing increases of 14.77% and 14.28%, respectively, relative to the control. Regarding TFC, Kal-SP at 1% and 7% exhibited significantly higher concentrations than the control, i.e., 28.57% and 22.86%, respectively. Additionally, plants treated with Kal-SC at 7% presented the highest antioxidant activity, with a 4.11% increase, significantly differing from all other treatments (Table 3).

The analysis of the total soluble sugar (TSS) dataset revealed that plants treated with both Kal-SC and Kal-SP at 7% did not differ from the control, unlike the other treatments. Regarding total starch (TS), the Kal-SP treatments (control, 1%, and 3%) did not differ from the control, nor did the Kal-SC-treated plants at 7%. For total carbohydrates (TCs), no statistical difference was detected for Kal-SP treatments; however, plants treated with Kal-SC at 3%, 5%, and 7% differed significantly from the control, with increases of 14.01%, 12.15%, and 13.56%, respectively (Table 4).

In the total protein (TP) analysis, the control group showed the highest content in both Kal-SP- and Kal-SC-treated plants, in contrast to the results observed for total amino acids (TAA). For TAA, the highest concentrations were found in plants treated with the algal bioinput produced in the State of São Paulo (i.e., Kal-SP at 1%, 3%, 5%, and 7%). Similarly, plants treated with Kal-SC at 3%, 5%, and 7%, followed by the 1% treatment, also exhibited significantly higher TAA contents than the control (*p* < 0.05). Overall, compared with the control, TAA levels in Kal-SP-treated plants increased by 18.04%, 25.97%, 32.18%, and 39.83% for the 1%, 3%, 5%, and 7% treatments, respectively. Even greater increases were observed in plants treated with Kal-SC, with increments of 22.93%, 57.02%, 49.79%, and 53.10% for the respective concentrations (Table 4).

**Figure 2:**
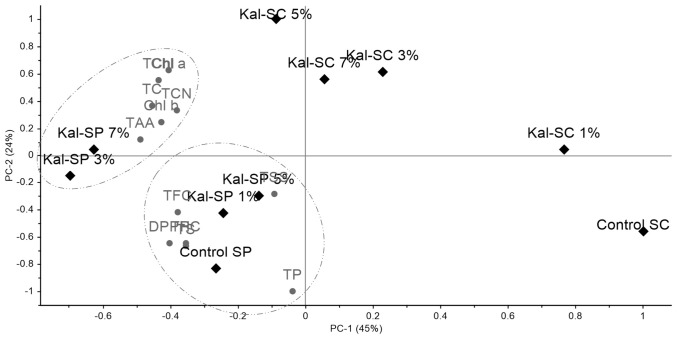
Principal component analysis (PC1 and PC2) calculated from the dataset of total contents of chlorophyll *a* (Chl *a*), chlorophyll *b* (Chl *b*), total chlorophyll (TChl), carotenoids (TCN), soluble sugars (TSS), starch (TS), carbohydrates (TC), phenolics (TPC), flavonoids (TFC), proteins (TP), amino acid (TAA) and antioxidant activity (DPPH assay) of basil plants treated with foliar application of *Kappaphycus alvarezii* biostimulants (1%, 3%, 5%, and 7%) cultivated in the locations of São Paulo (SP) and Santa Catarina (SC), Brazil.

In the PCA, 69% of the total variance was explained by PC1 and PC2, with PC1 accounting for 45% of the variance in the descriptive model. Two clusters were identified, both consisting of Kal-SP treatments. The Kal-SC samples were separated and not grouped by variable. The first cluster was formed by Kal-SP at 3% and 5%, due to their contents in pigments (e.g., Chl *a*, Chl *b*, TChl, and TCN), TC, and TAA. Meanwhile, the Kal-SP 1% and 5% treatments were grouped regarding their TPC, TFC, DPPH, TSS, TS, and TP (Figure 2; Table S2, Supplementary Material).

### 2.4. Essential Oil Yield and Composition

**Figure 3:**
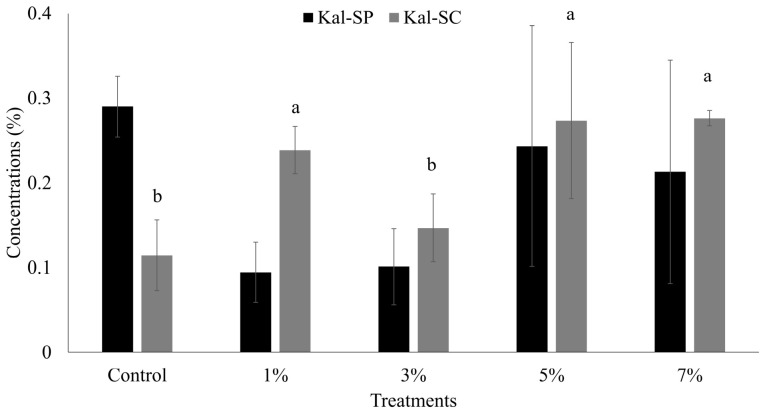
Essential oil yield of basil plants treated with foliar application of *Kappaphycus alvarezii* biostimulant (1%, 3%, 5%, and 7%) cultivated in the locations of São Paulo (SP) and Santa Catarina (SC), Brazil. Statistical analysis performed separately for each group (i.e., Kal-SP and Kal-SC) using the Scott–Knott test (*p* < 0.05). Distinct letters indicate statistically significant differences (observed only for Kal-SC).

The yield analysis of the essential oil fraction of basil plants revealed a range of contents for Kal-SP samples from 0.09% to 0.29% (0.09–0.29 mg/100 g), somewhat similar to the findings noted for Kal-SC samples, i.e., from 0.11% to 0.28% (0.11–0.28 mg/100 g). No statistically significant differences were observed among the treatments for Kal-SP. The Kal-SC plants treated with the algal biostimulant at 1%, 5%, and 7% showed statistically significant differences in essential oil content compared with the control, with increases of 108.77%, 139.47%, and 142.11%, respectively (Figure 3).

The GC-MS profile of the basil essential oil identified 22 compounds, including cadinane-type sesquiterpenes observed across four retention times, as well as one peak associated with cadinol/muurolol and another guaiane-type sesquiterpene (Table 5; Figure S3, Supplementary Material). Considering the complete chromatograms, the total peak area percentage ranged from 68.04% to 80.09% among the samples.

The identified compounds included the non-oxygenated monoterpene α-pinene or β-pinene (which could not be clearly distinguished based on the analytical method), four oxygenated monoterpenes (1, 8-cineole, β-terpineol, terpinen-4-ol, and α-terpineol), the phenylpropanoid eugenol, nine sesquiterpene hydrocarbons (α-farnesene, β-farnesene, (*Z*,*Z*)-α-farnesene, α-humulene, germacrene D, β-caryophyllene, epi-bicyclosesquiphellandrene, a cadinane-type sesquiterpene, and a guaiane-type sesquiterpene), two oxygenated sesquiterpenes (spathulenol and cadinol/muurolol), one diterpene (phytol), and one long-chain compound (hexahydrofarnesyl acetone).

Of the 22 compounds identified, 12 were detected in all samples, while the remaining compounds were absent in one or more samples (Table 5). However, the major constituents remained the same across all samples. The most abundant compounds were epi-bicyclosesquiphellandrene (13.94% to 24.83%), followed by *α*-farnesene (9.79% to 14.82%) and eugenol (6.97% to 14.91%). In minor amounts, a cadinane-type sesquiterpene (4.92% to 7.19%, retention time 18.45–18.59), followed by terpinen-4-ol (0% to 5.75%), phytol (1.39% to 5.46%), and another cadinane-type sesquiterpene (2.19% to 3.96%, retention time 21.99–22.12) were detected.

Upon applying statistical analysis to the major compounds (Table 6), i.e., epi-bicyclosesquiphellandrene, α-farnesene, and eugenol, a distinction was observed between the results from the Kal-SP- and Kal-SC-treated plants. For Kal-SP, the control showed higher levels of α-farnesene and eugenol than some biostimulant-treated samples, particularly the 1%, 5%, and 7% treatments for α-farnesene, and the 5% and 7% treatments for eugenol. In contrast, for epi-bicyclosesquiphellandrene, treatments at 5% and 7% increased the content of this major compound by 70.02% and 44.17%, respectively. For Kal-SC, the opposite trend was observed: epi-bicyclosesquiphellandrene showed higher contents in the control, with no differences compared with the 1% and 7% treatments, but differing from the other treatments. For α-farnesene, the 3% and 5% treatments were higher than the control, with increases of 27.41% and 20.57%, respectively. For eugenol, Kal-SC treatments at 1%, 3%, and 5% showed increases of 55.16%, 39.84%, and 50.46%, respectively, compared with the control.

**Figure 4:**
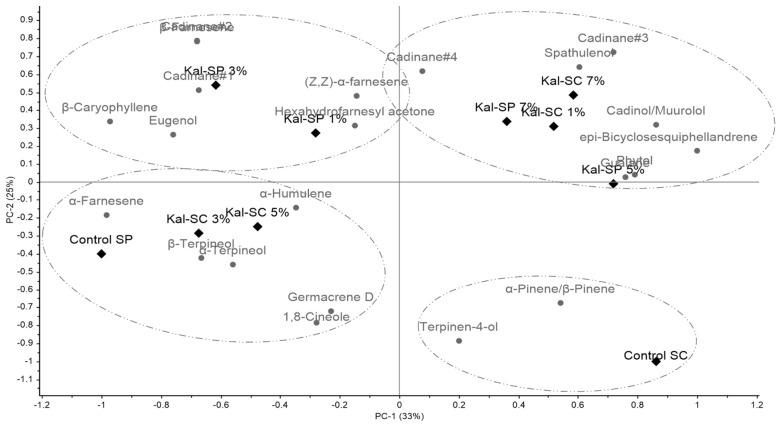
Principal component analysis (PC1 and PC2) of the GC-MS profiles of the essential oil constituents extracted from basil leaves treated with foliar applications of *Kappaphycus alvarezii* biostimulants (1%, 3%, 5%, and 7%) and cultivated in São Paulo (SP) and Santa Catarina (SC), Brazil.

In a second approach, PCA was applied to the dataset of metabolite contents identified in basil’s essential oil fraction. PC1, PC2, and PC3 explained 72% of the dataset’s total variance, and four distinct groups were formed (Figure 4; Figure S4, Supplementary Material; Table S2, Supplementary Material). The samples of Kal-SC control plants clustered (PC1+, PC2−) due to their terpinen-4-ol and α-pinene/ß-pinene contents. In their turn, the SP control- and the Kal-SC 3% and 5% treated plants grouped in PC1−/PC2− according to their α-farnesene, α-terpineol, ß-terpineol, α-humulene, germacrene D, and 1,8-cineole concentrations. The Kal-SP 1% and 3% samples clustered (PC1−, PC2+) as a result of their (*Z*, *Z*)-α-farnesese, ß-farnesese, hexahydrofarnesyl acetone, β-caryophyllene, eugenol, and two cadinane-type sesquiterpenes contents. Finally, the Kal-SP 5% and 7% treatments, together with the Kal-SC 1% and 7% treatments, were grouped in PC1+/PC2+ based on their contents of epi-bicyclosesquiphellandrene, cadinol/muurolol, spathulenol, phytol, two cadinane-type sesquiterpene compounds, and one guaiane-type sesquiterpene compound.

**Figure 5:**
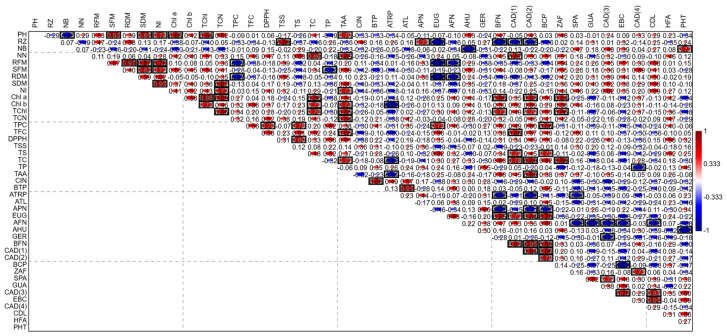
Pearson correlation analysis of morphological and biochemical parameters, as well as essential oil constituents, extracted from basil leaves treated with foliar applications of *Kappaphycus alvarezii* biostimulants (1%, 3%, 5%, and 7%) and cultivated in São Paulo (SP) and Santa Catarina (SC), Brazil. NI—number of inflorescences; PH—plant height; RL—root length; NB—number of branches; NN—number of nodes; RFM—root fresh mass; SFM—shoot fresh mass; RDM—root dry mass; SDM—shoot dry mass; Chl *a*—chlorophyll *a*; Chl *b*—chlorophyll *b*; TChl—total chlorophyll; TCN—carotenoids; TSS—soluble sugars; TS—starch; TC—carbohydrates; TPC—phenolics; TFC—flavonoids; TP—proteins; TAA—amino acids; CIN—1,8-cineole; BTP—β-terpinene; ATRP—α-terpinene; ATL—α-Terpineol; APN—α-Pinene/β-Pinene; EUG—eugenol; AFN—α-farnesene; AHU—α-humulene; GER—germacrene; BFN—β-farnesene; CAD—cadinane (group); BCP—β-caryophyllene; ZAF—(Z,Z)-α-farnesene; SPA—spathulenol; GUA—guaiane; EBC—epi-bicyclo (sesquiterpene type); CDL—cadinol; HFA—hexahydrofarnesyl acetone; PHT—phytol.

Pearson correlation analysis (Figure 5) of the morphological, biochemical, and essential oil composition datasets revealed both positive (0.63 to 0.98) and negative (−0.64 to −0.88) correlations. A focused analysis among the parameters showed that PH correlated positively with SFM, SDM, NI, Chl a, TChl, and TAA. RZ was correlated with TSS and α-Pinene/β-Pinene (APN). NB was correlated with phytol (PHT), while NN correlated with TC, TAA, and a cadinane (CAD) compound. SDM correlated with NI and TCN, and NI correlated with TCN and TAA. Pigments were strongly intercorrelated, and Chl a and TChl also showed positive correlations with TC and with β-farnesene (BFN), a CAD compound, and (Z,Z)-α-farnesene (ZAF). TPC clustered positively with DPPH, TS, eugenol (EUG), and β-caryophyllene (BCP). TFC was correlated with TAA and a CAD compound. TS correlated with a CAD compound and BCP, while TC correlated with TAA, two CAD compounds, and ZAF.

In contrast, fewer negative correlations were observed. RZ was negatively associated with EUG, BFN, and two CAD compounds, whereas NB was negatively associated with α-humulene (AHU). RFM and RDM were negatively correlated with TPC, EUG, and α-farnesene (AFN). SFM was negatively correlated with TP. Chl b, TC, and TAA were negatively associated with α-terpinene (ATRP). TP was negatively correlated with a CAD compound (Figure 5).

### 2.5. Rhizosphere Microbiome

**Figure 6:**
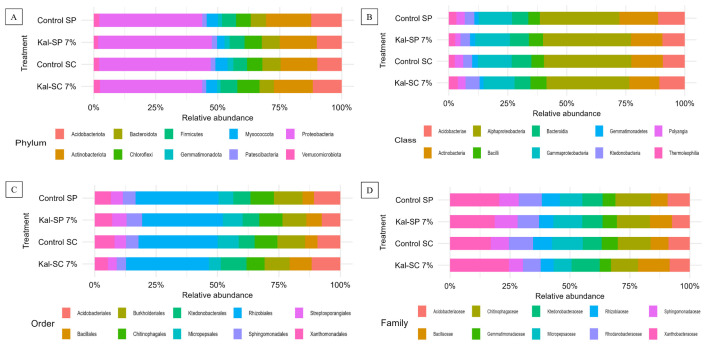
Predominant (relative abundance) phyla (**A**), classes (**B**), orders (**C**), and families (**D**) in the rhizosphere of basil plants sprayed with *Kappaphycus alvarezii* biostimulant at 7%, according to the origin of that bioproduct, i.e., São Paulo (Kal-SP) and Santa Catarina (Kal-SC) States, Brazil.

The alpha diversity indexes of prokaryotic communities present in basil rhizosphere treated with 7% *K. alvarezii* (Kal-SP and Kal-SC) showed no significant differences compared to their controls (Table S3, Supplementary Material). Forty-one phyla were identified in the basil rhizosphere. Among these, Proteobacteria were the most prevalent in all treatments, followed by Actinobacteriota and Acidobacteriota (Figure 6A). At the class level, 114 classes were identified. The most represented class was Alphaproteobacteria, followed by Gammaproteobacteria and Actinobacteria (Figure 6B). In terms of orders, 277 orders were identified, with Rhizobiales being the most frequent, followed by Burkholderiales and Acidobacteriales (Figure 6C). These analyses also identified 439 families, with Xanthobacteriaceae being the most significant, followed by Chitinophagaceae (Figure 6D).

Statistical analysis using non-parametric methods was applied to each taxonomic category with relative abundance above 1% (Table S4, Supplementary Material), considering separately each state where *K. alvarezii* was collected (i.e., Kal-SP vs. Control SP and Kal-SC vs. Control SC). Based on the SIMPER analysis, the contribution to dissimilarity between treatments and controls was higher at the family level (15.6% for SP and 20.8% for SC; Table S4, Supplementary Material). At this taxonomic level, in SP samples, *Microscillaceae* showed a higher relative abundance under the biostimulant treatment (0.95%) compared to the control (0.62%). In SC samples, the control exhibited higher relative abundances of *Micropepsaceae* and *Caulobacteraceae* (4.04% and 1.51%, respectively) than Kal-SC 7% (2.08% and 0.91%, respectively). Conversely, *Bacillaceae* was more abundant under the biostimulant treatment (3.88%) than in the control (2.34%).

**Figure 7:**
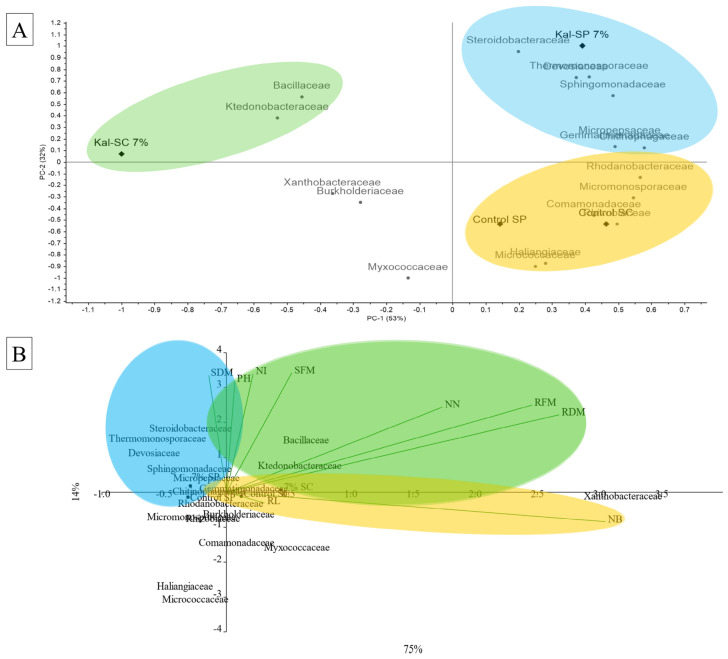
Principal component analysis (**A**) and canonical correspondence analysis (**B**) of the dataset for predominant families (SIMPLER test) in the rhizosphere of basil plants sprayed with a 7% biostimulant of *K. alvarezii* from São Paulo (Kal-SP) and Santa Catarina (Kal-SC), Brazil.

PCA explained 85% of the total variance (PC1 and PC2) of the microbiome dataset and revealed three distinct groupings. Control samples clustered in PC1+/PC2-, whereas biostimulant-treated plants were separated from both the controls and each other. Thus, Kal-SP 7% samples were positioned in proximity to families such as *Chitinophagaceae*, *Micropepsaceae*, *Gemmatimonadaceae*, *Sphingomonadaceae*, *Thermomonosporaceae*, *Steroidobacteraceae*, and *Devosiaceae*, indicating that these taxa contributed most to the observed variation. In contrast, Kal-SC 7% samples were associated primarily with *Bacillaceae* and *Ktedonobacteraceae*. Control samples were positioned closer to *Rhodanobacteraceae*, *Rhizobiaceae*, *Comamonadaceae*, *Micromonosporaceae*, *Haliangiaceae*, and *Micrococcaceae* (Figure 7A; Table S2, Supplementary Material).

Canonical correspondence analysis (CCA) indicated a clear separation of treatments into distinct ordination quadrants (Figure 7B). The Kal-SC 7% treatment was associated with more plant growth parameters (NI, SFM, NN, RFM, and RDM) and clustered with *Bacillaceae* and *Ktedonobacteraceae*. In contrast, the Kal-SP 7% treatment was associated with the same families identified in the PCA and aligned primarily with SDM. Control SC plants were associated with RL and NB, whereas control SP plants showed no morphological variable associations, only with rhizosphere bacteria (Rhodanobacteriaceae, *Burkholderiaceae*, *Micromonosporaceae*, *Rhizobiaceae*, *Comamonadaceae*, *Haliangiaceae*, and *Micrococcaceae*).

## 3. Discussion

### 3.1. Morphological and Biochemical Parameters

Both morphological and biochemical responses differed following application of Kal-SP and Kal-SC biostimulants. These results indicate that, in addition to concentration, the origin of the *K. alvarezii* biomass used to prepare the biostimulant influences basil plants’ responses, particularly their biochemical traits. The two biostimulants were previously characterized in terms of composition [13], revealing quantitative discrepancies among their constituents. Kal-SP contained a higher total phenolic content (~161.11%) than Kal-SC, whereas the latter showed superior content of total carotenoids (~60.95%). No significant differences were detected between the two extracts for total flavonoids, carbohydrates, soluble sugars, starch, and protein. 1D and 2D NMR spectroscopy analysis identified 17 metabolites, of which 11 (alanine, betaine, glutamate, glycine, isoleucine, methionine, phenylalanine, taurine, tyramine, uracil, and valine) were more abundant in Kal-SP. At the same time, six (4 aminobutyrate, acetate, formate, lactate, leucine, and succinate) were more abundant in Kal-SC. Analysis of macro and micronutrient contents in samples of the source macroalga revealed that six elements (Cu, Mn, Ca, Mg, K, and N) were detected in higher mean concentrations in Kal-SP. In comparison, four elements (B, Zn, P, and S) were superior in Kal-SC [13]. Such compositional discrepancies are strongly influenced by environmental factors (e.g., salinity and water temperature) that affect algal physiology and biochemical composition [11,17] and, consequently, can modulate plant responses, as observed in this research.

Comparisons with previous studies demonstrate that higher concentrations, such as 5% and 7%, within the tested range (1–7%) commonly elicit morphophysiological improvements. However, evidence from other studies indicates that concentrations above 7% may also provide beneficial effects. While increases in root and leaf dry mass were recorded in rice with 5% and 10% extracts [18], similar growth and productivity results were obtained with doses up to 15% [19]. Similarly, improvements in rice yield were validated at 7.5% [20]. These benefits are not restricted to cereals; in potato crops, plants treated with the algal biostimulant at 5% and 7.5% also enhanced tuber quality and productivity [21]. However, despite the promising results obtained with the use of *K. alvarezii* biostimulant, it is important to highlight that each study has been conducted with different plant species, under distinct application methods and environmental conditions; therefore, dose–response relationships should always be evaluated on a case-by-case basis.

Regarding biochemistry, although maize under water deficit treated with a 2.5% *K. alvarezii* extract showed substantial metabolic adjustments involving carbohydrates, lipids, and hormones [22], other findings highlighted different mechanisms. Specifically, increased enzymatic activity and overall yield were observed in maize when using higher concentrations, e.g., 5% and 10% biofertilizer [23]. Overall, these findings suggest that concentrations above 7% may also be of interest for basil plants, both morphologically and biochemically, warranting further investigation in future studies.

In a related study by the same research group [24], basil grown hydroponically with the same biostimulants investigated herein showed that Kal-SP at 3% increased plant height and root length, while Kal-SC at 3% and 5% increased node number, and Kal-SC at 5% promoted higher root dry mass. Biochemically, Kal-SP at 5% increased pigment levels (Chl *a*, Chl *b*, TChl, and TCN), TSS (also at 1%, 3%, and 7%), TS (also at 3% and 7%), TC (also at 3%), and TAA (also at 7%). Kal-SC increased Chl *a* and TChl at 1% and 7%, and TCN at 1%, 3%, and 7%. TSS, TS, and TC were elevated at Kal-SC concentrations of 5% and 7%. Kal-SC at 1% and 5% increased TPC.

In hydroponically grown peppermint treated with the same biostimulants, biochemical parameters increased, although some growth variables showed no significant differences. For instance, TSS increased by 3% in treatments with both Kal-SP and Kal-SC relative to the control. Kal-SP at 1%, 5%, and 7%, and Kal-SC at 5% also raised TSS compared to control plants. TS increased with Kal-SP at 5% and with Kal-SC at 3%, 5%, and 7%, as TC increased with Kal-SP at 5% and 7% and with Kal-SC at 3% and 5%. Finally, TFC increased with Kal-SP and Kal-SC at 1% [25].

Taken together, these findings indicate that *K. alvarezii*-derived biostimulants can differentially affect crop species, producing variable morphological and biochemical effects depending on the extract’s origin, concentration, and crop system. The type of cultivation system (soil vs. hydroponic), plant species, and environmental context are important modulators of response. Nonetheless, the evidence supports the potential utility of *K. alvarezii* extracts as agricultural inputs, a conclusion consistent with recent reviews on the biostimulant of this seaweed species [13,14].

### 3.2. Essential Oil

For the essential oil analysis, epi-bicyclosesquiphellandrene, α-farnesene, and eugenol were the major constituents detected in the basil samples. Epi-bicyclosesquiphellandrene, although previously reported in *O. basilicum* [26,27], remains relatively underexplored in the literature, particularly with respect to its biological properties. α-farnesene, in turn, is a compound whose ecological functions are widely documented and classically associated with plant responses to biotic stress, especially herbivory. As a volatile organic compound, α-farnesene acts as an inter-plant alarm signal, playing a role in tritrophic signaling by attracting natural enemies of herbivores and thereby contributing to the plant’s indirect defense [28,29]. Finally, eugenol, commonly reported in *O. basilicum* [30], is one of the most agronomically, pharmaceutically, and industrially relevant constituents of the essential oil fraction. This relevance is linked to its pharmacological properties, including antimicrobial, anticancer, antioxidant, anti-inflammatory, and analgesic activities [31].

Considering the statistical analysis of these major compounds, distinct response patterns were observed between plants treated with the *K. alvarezii* biostimulant produced in the SP and SC states, indicating that the geographic origin of the macroalgal biomass used to produce the biostimulant is a determining factor in modulating the plant’s specialized metabolism. The opposite trend observed between Kal-SP and Kal-SC further supports the hypothesis that differences in the chemical composition of the biostimulants [13] led to divergent elicitation profiles.

However, PCA shows that the differential effect of the bioproduct in basil plants was not determined solely by the distinct origin thereof (as reflected by the clustering of Kal-SC 3% and Kal-SC 5%, as well as Kal-SP 1% and Kal-SP 3%). It also depends on the biostimulant concentration used. In fact, 7 treated plants were grouped in the same quadrant, regardless of the biostimulant origin (i.e., Kal-SP and Kal-SC 7%). This observation has relevant practical implications: the combined selection of the biostimulant’s origin and the applied dose enables the targeted production of specific compounds of interest in basil essential oil, opening perspectives for objective-driven phytochemical management in agroindustry settings.

Additionally, by applying Pearson correlation analysis, it was found that there is a strong integrative relationship among morphological traits, biochemical responses, and essential oil composition, suggesting that growth performance, primary metabolism, and secondary metabolite biosynthesis are closely interconnected.

### 3.3. Rhizosphere Microbiome

The application of the *K. alvarezii*-based biostimulant did not result in significant differences in alpha-diversity indices of prokaryotic communities in the basil rhizosphere, suggesting that the treatment did not induce detectable changes in microbial richness or evenness. This finding is consistent with maintaining community structural stability, a desirable feature in sustainable management strategies that modulate the microbiota without causing pronounced disturbances to the resident community [32,33]. However, despite the stability observed in alpha diversity indices, shifts in the structure and taxonomic composition of microbial communities were detected, which may ultimately have functional implications for the rhizosphere, including changes in nutrient cycling, recruitment of plant growth-promoting microorganisms, and pathogen suppression, ultimately influencing plant performance [34,35,36].

Multivariate analyses (PCA and CCA) indicated a consistent separation between biostimulant-treated samples and control ones, suggesting that the use of the *K. alvarezii* bioproduct is associated with shifts in the structure of the rhizosphere microbial community. These patterns indicate that the observed variation is not random, but reflects a community reorganization potentially linked to the presence of the biostimulant and plant–soil system responses. Similar findings have been reported in studies where biostimulant application alters rhizosphere microbial structure, often in association with changes in root exudation and plant physiological status [37,38].

A particularly relevant aspect was the differential response between biostimulants derived from distinct origins (SP and SC). The Kal-SC treatment showed a more direct association with plant growth parameters (NI, SFM, NN, RFM, and RDM) and a narrower set of associated bacterial families, suggesting a more selective enrichment of specific microbial groups. In contrast, the Kal-SP treatment was associated with a broader range of bacterial families, indicating a more diffuse modulation of the community. These differences may be related to variations in the chemical and bioactive composition of *K. alvarezii* biomass [13], leading to distinct effects on the soil microbiome.

The strong association observed between the Kal-SC 7% treatment and the bacterial families *Bacillaceae* and *Ktedonobacteraceae*, as well as plant growth parameters (NI, SFM, NN, RFM, and RDM), suggests a functional interaction between microbiome structure and plant performance. Although direct causality cannot be inferred, these groups may be linked to ecologically relevant functions. Members of the *Bacillaceae* family are commonly described as copiotrophic microorganisms with rapid responses to labile carbon inputs and well-established plant growth-promoting traits, including phytohormone production, nutrient solubilization, and antagonistic activity against pathogens [39].

*Ktedonobacteraceae*, in contrast, has been associated with the degradation of complex organic compounds and soil carbon cycling, potentially contributing indirectly to nutrient availability [40,41]. The co-occurrence of these groups suggests a potential functional balance between copiotrophic microorganisms and taxa involved in the decomposition of more recalcitrant organic matter, which may promote coupling between the breakdown of complex substrates and the rapid assimilation of intermediate products (cross-feeding) [42]. This functional arrangement can enhance the efficiency of carbon and nutrient cycling in the soil, supporting a more continuous release of resources and contributing to microbiome functional stability. Consequently, this balance may help sustain a rhizosphere environment more conducive to plant growth.

A possible mechanistic explanation involves the sulfated polysaccharides present in *K. alvarezii*, particularly carrageenans, which may act as complex carbon sources and selective substrates for microorganisms capable of degrading sulfated polymers [43]. The introduction of these compounds into the soil could favor taxa harboring enzymatic systems, such as sulfatases and specialized carbohydrate-active enzymes, while also supporting copiotrophic populations by releasing intermediate degradation products [44]. Additionally, oligosaccharide fragments derived from these sulfated polysaccharides may function as bioactive molecules, potentially modulating plant physiological responses and altering root exudation patterns [45]. This process could enhance the selective recruitment of specific microbial groups, reinforcing the functional structuring of the rhizosphere microbiome.

In contrast, the families associated with the Kal-SP 7% treatment (*Chitinophagaceae*, *Micropepsaceae*, *Gemmatimonadaceae*, *Sphingomonadaceae*, *Thermomonosporaceae*, *Steroidobacteraceae*, and *Devosiaceae*) represent a functionally heterogeneous assemblage with partial niche overlap. These taxa are frequently linked to the degradation of complex organic polymers, including chitin and aromatic compounds, nutrient cycling, and adaptation to environments with lower availability of labile carbon [46,47].

Although these families do not necessarily co-occur as a stable consortium, they share metabolic strategies consistent with more complex substrate utilization, including specialized degradation pathways and efficient resource use under nutrient-limited conditions. This pattern suggests that the Kal-SP 7% treatment promoted a less selective and more diffuse reorganization of the microbial community, favoring multiple functional guilds rather than enriching taxa more directly associated with plant growth promotion.

From an applied perspective, the results indicate that seaweed-based biostimulants can influence plant growth and are associated with shifts in soil microbial community structure. The maintenance of alpha diversity indices suggests that this modulation occurs without detectable changes in microbial richness and evenness, a desirable feature in sustainable strategies that aim to manage the microbiome without inducing major disturbances to the resident community.

Furthermore, the findings indicate a reorganization of the rhizosphere microbial community associated with the application of *K. alvarezii*, with patterns dependent on the origin of the biomass (SP vs. SC). These results highlight the importance of environmental conditions and biomass processing in shaping the chemical composition of the extracts and, consequently, their effects on the plant–soil system. In this context, standardization of such inputs is critical to ensuring consistent and predictable agronomic performance.

## 4. Materials and Methods

### 4.1. Sample Collection

Samples of *K. alvarezii* (red and green strains) were collected from the Fisheries Institute at the Research and Development Center of the North Coast in Ubatuba, São Paulo (SP) (23°27′07″ S, 45°02′49″ W—Southeast Region), as well as from a marine farm located in the municipality of Florianópolis, Santa Catarina (SC) (27°42′32.724″ S, 48°33′35.5″ W—South Region), Brazil. Samples of *K. alvarezii* were collected simultaneously in SP and SC to ensure consistency regarding seasonality (summer) and duration of cultivation (Figure S1, Supplementary Material).

### 4.2. Biostimulant Extraction

Before extraction, the collected algal biomass was washed with chlorinated water to remove salt, impurities, and epiphytic organisms. The red and green strains of *K. alvarezii* were then weighed separately to ensure consistency in the extraction process, using equal proportions of fresh biomass (500 g) for biostimulant production.

The extraction process was carried out using an industrial blender (Metvisa^®^, Brusque, Santa Catarina, Brazil; 800 W; 18,000 rpm) at room temperature. The macroalgae were first washed, cut into small pieces, and then homogenized in the blender for a standardized period to obtain a uniform slurry (5 min). The resulting material was subsequently filtered using qualitative filter paper (Unifil^®^, Curitiba, Paraná, Brazil; 80 g m^−2^; 0.16 mm thickness) under gravity filtration to remove coarse particles and obtain a clarified extract. The biostimulant samples (100% concentration) were stored at −20 °C until analysis. To differentiate the *K. alvarezii* biostimulants from São Paulo and Santa Catarina, the bioproduct samples were labeled as Kal-SP and Kal-SC, respectively.

### 4.3. Cultivation System and Treatments

The cultivation of basil plants (*Ocimum basilicum*) was conducted at the Ecological Park of Cidade das Abelhas of the Federal University of Santa Catarina (UFSC), located in Florianópolis, Southern Brazil (27°53′175.61″ S, −48°50′32.08″ W). The cultivation lasted 80 days, from 27 January to 17 April 2025.

Before the experiment began, a soil analysis (Table S1, Supplementary Material) was performed, and soil correction was carried out. Limestone was applied once at approximately 2.5 t ha^−1^ to correct soil acidity to 6, two months before seedling transplantation. Basil seedlings (30-day-old) were purchased from a specialized nursery and transplanted into the field ten days after correcting the soil. Basal fertilization consisted of 0.3 t ha^−1^ of NPK (10:10:10) at planting, followed one month later by a topdressing of 0.3 t ha^−1^ of a mixed fertilizer (Dimy^®^, Cajamar, São Paulo, Brazil) containing the same NPK ratio plus calcium (5.4%), magnesium (2.5%), and sulfur (1.0%).

A completely randomized block design was used, with two blocks of nine plants each, resulting in a total of 18 plants per treatment. Three plants were arranged per row, with a 0.3 m separation between plants. Ten treatments were considered as follows: two controls with distilled water (applied in both blocks where the Kal-SP and Kal-SC treatments were conducted); Kal-SP at concentrations (*v*/*v*) of 1%, 3%, 5%, and 7%; and Kal-SC at concentrations of 1%, 3%, 5%, and 7%. Weekly spraying was carried out, totaling 10 applications along the cultivation cycle between 10 February and 13 April 2025. Foliar applications were performed until runoff; therefore, the spray volume increased progressively with plant growth and canopy development throughout the cultivation period.

### 4.4. Environmental Data

Environmental data for the period between 27 January and 17 April 2025 were kindly provided by the Agricultural Research and Rural Extension Company of Santa Catarina (EPAGRI) through the Center for Environmental Resources and Hydrometeorology Information (CIRAM). Daily data on precipitation (mm), humidity (%), and air temperature (°C) were analyzed to provide an overview of the environmental conditions during the experimental period [48].

### 4.5. Basil Morphological Analysis

The analysis of basil plant flowering was performed 48 days after the start of the experiment, with subsequent evaluations at 62 and 76 days. All plants were checked for flowers.

At the end of the experiment, all plants were analyzed for the following parameters: plant height (cm), number of nodes (n), root length (cm), shoot fresh weight (g), and root fresh weight (g). Plant height and root length were measured using a ruler (n = 180). Weighing was performed using an analytical balance (Shimadzu AUY 220). To obtain the dry weight (g) of roots and aerial parts, samples were dried in a forced-air oven with air circulation and renewal, equipped with a fan for air movement and a temperature controller (DeLeo DL—AF, Porto Alegre, RS, Brazil), at 40 °C until constant weight was achieved, which occurred after approximately 7 days.

For root weight, plants from all treatments were used (n = 180), while for the determination of the aerial part weight, three plants from the first row of each block were collected, totalizing six plants per treatment (n = 60). This subdivision was necessary to ensure sufficient and standardized plant material for both biomass measurements and essential oil extraction. The remaining plants were prepared for essential oil extraction. For that, the aerial part biomass was dried in darkness in an air-conditioned room at 20 °C, with a timer-controlled fan running for 2 h every 4 h.

### 4.6. Basil Biochemical Analysis

For biochemical analysis, four fully expanded mature leaves were collected per plant from each treatment in both blocks, positioned immediately after the first node from the apex. Sampling was performed in the early morning (06:00 h) to minimize potential diurnal variation in pigment and metabolite levels, one day prior to plant harvesting for morphological analysis. A total of 36 leaves per treatment were collected. The leaves were placed in properly labeled Ziplock plastic bags, transported in an ice bath to the laboratory, and processed immediately for biochemical analysis. Prior to analysis, the central midrib was removed to ensure sample uniformity. For each treatment, six biological replicates (three per block) were included, and each sample was additionally analyzed in triplicate, resulting in a total of 18 analytical determinations per treatment. All samples were subsequently stored in Falcon tubes at −20 °C until further analysis.

Analysis of total, *a*, and *b* chlorophylls, as well as total carotenoids [49,50], was conducted on the same day the leaves were collected, due to the potential degradation of these compounds. Following this, the total contents of phenolic compounds [51], flavonoids [52], antioxidant activity using the 2,2-diphenyl-1-picrylhydrazyl radical (i.e., DPPH assay) [53], soluble sugars and starch [54], carbohydrates [55], protein [56], and amino acids [57] were determined. To perform the analyses, the leaves were initially macerated with liquid nitrogen to ensure appropriate maceration and release of compounds.

### 4.7. Extraction and Characterization of Essential Oil

For the essential oil analysis, dried leaves were separated from stems and inflorescences, ground, and stored in the dark at room temperature (~25 °C). Essential oil extraction was performed in triplicate (n = 3) for each treatment by mixing 6 g of leaf biomass with 120 mL of distilled water. The extraction was carried out using a Clevenger apparatus for 6 h. The system was maintained under hydrodistillation conditions, with the heating mantle set at 260 °C to ensure continuous boiling of the water and steam distillation of the essential oil. Essential oil yield was expressed as a percentage (%) and as mg per 100 g of leaf dry weight.

For the chemical characterization of the essential oils, the samples (n = 3) were diluted in hexane (1: 10, *v*/*v*) before analysis by gas chromatography coupled to mass spectrometry (GC–MS, Hewlett Packard 6890/MSD5973). GC-MS analyses were performed using a DB-5 capillary column (30 m × 0.32 mm i.d., 0.50 μm film thickness). The oven temperature program began at 60 °C and was ramped at 3 °C/min to 246 °C. The injector temperature was set to 220 °C. A split injection mode was applied (i.e., split ratio = 1:20), and Helium was used as the carrier gas (flow rate 1.0 mL/min, 56 kPa), resulting in a total run time of 62 min. The interface temperature was maintained at 240 °C, and the mass spectrometer was operated at an electron ionization (EI) energy of 70 eV [58]. The data were expressed as a percentage of area (%).

GC-MS was used to identify compounds by comparing the acquired mass spectra with the Wiley library and evaluating the experimentally determined linear retention indices against values reported in the literature (NIST). Final compound confirmation was supported by additional cross-referencing with multiple published studies [26,59,60,61,62,63,64,65].

### 4.8. Soil Microbiome Analysis

Prokaryotic community characterization was performed on treatments showing the greatest effects on basil growth (i.e., Kal-SP 7% and Kal-SC 7%) and the control, following the procedures described by de Sousa et al. [34]. For this purpose, rhizosphere soil was collected after carefully excavating the plants to preserve the entire root system for morphological analysis. Before separating shoots and roots, each plant was placed on a sterile sieve and gently shaken to detach the soil closely adhered to the root surface, which was considered rhizosphere soil. The collected samples were immediately stored at −80 °C until analysis. No bulk soil (non-rhizosphere soil) control was included in the experimental design.

Total genomic DNA was extracted from 200 mg of soil using the PureLink™ Microbiome DNA Purification Kit (Invitrogen, Waltham, Massachusetts, USA). The V3–V4 region of the 16S rRNA gene was amplified by PCR using primers 341F and 806R, and amplicons were sequenced on an Illumina MiSeq platform (Illumina Inc., San Diego, CA, USA) according to the manufacturer’s instructions. Raw reads (FASTQ format) were processed using QIIME 2 (v.2020.8), with the DADA2 plugin for quality filtering, chimera removal, and amplicon sequence variant (ASV) inference. Taxonomic assignment was performed using the q2-feature-classifier plugin against the SILVA v138 database (99% similarity). Subsequent analyses were conducted in R (v. 4.0) using the Phyloseq and Vegan packages. Sequencing depth was assessed using rarefaction curves.

### 4.9. Statistical Analysis

For the statistical analyses, all data were subjected to normality testing (Shapiro–Wilk test) and homogeneity of variance testing (Levene’s test) to guide the selection of appropriate statistical procedures. Based on these assumptions, morphological and biochemical data that met normality and homoscedasticity criteria were analyzed using the Scott–Knott test, whereas flowering-related variables that did not satisfy these assumptions were evaluated using the non-parametric Kruskal–Wallis test. Subsequently, Dunn’s test was applied for multiple comparisons. All analyses were conducted using the RStudio suite (v. 4.3.2).

For the analysis of morphological data, the Scotty and Knott test (*p* < 0.05) was used for mean comparisons at a 5% error probability. For biochemical analyses and essential oil yield, the Scott–Knott test was used without data transformation (*p* < 0.05). All data were analyzed using AgroEstat software (v. 1.1.0.712). Morphological and biochemical parameters, as well as essential oil composition, were subjected to principal component analysis (PCA) using the singular value decomposition (SVD) algorithm. PCA was performed using Unscrambler^®^ X software (v. 10.4).

For the statistical analysis of prokaryotic communities, both alpha and beta diversity, as well as similarity profiles (SIMPER) analysis, were evaluated using Past software (v. 4.17). Dual comparisons between treatment and control groups were conducted using the Kruskal–Wallis test (*p* < 0.05) because the data were non-parametric. Canonical correspondence analysis (CCA) was performed to identify prokaryotic groups associated with basil morphological parameters. Additionally, these morphological and biochemical parameters, essential oil yields, and prokaryotic communities were subjected to principal component analysis (PCA) using the singular value decomposition (SVD) algorithm. The PCA was performed using the Unscrambler^®^ X software (v. 10.4).

## 5. Conclusions

The foliar application of *K. alvarezii* extracts promoted positive morphophysiological responses in *O. basilicum*, with biostimulant effectiveness strongly dependent on both extract origin and concentration, particularly at 5% and 7%. In addition to improving plant growth and productivity, the extracts directionally modulated the chemical composition of basil essential oil, altering the relative abundance of specific terpenoid compounds according to treatment and biomass origin.

Changes in rhizosphere microbial community structure and taxonomic composition occurred without significant losses in alpha diversity, indicating functional modulation while maintaining ecological stability. The enrichment of bacterial groups associated with plant growth promotion further suggests an interaction between biostimulant application and rhizosphere reorganization. Overall, the results demonstrate that compositional differences associated with biomass origin directly influence plant and microbiome responses, highlighting the importance of extract standardization to ensure consistent agronomic performance.

## Figures and Tables

**Table 1:** Mean value and standard deviation (±) of the number of inflorescences (n) of basil plants treated with foliar application of *Kappaphycus alvarezii* biostimulant (1%, 3%, 5%, and 7%) produced in the States of São Paulo (Kal-SP) and Santa Catarina (Kal-SC), Brazil.

Treatments	First Count (n.)	Second Count (n.)	Third Count (n.)
Control SP	0.61 ± 0.85 b	9.00 ± 7.44 d	30.50 ± 17.68 b
Kal-SP 1%	0.78 ± 0.73 b	9.22 ± 5.64 d	34.83 ± 18.46 b
Kal-SP 3%	1.11 ± 0.96 b	13.33 ± 8.44 c	39.22 ± 20.53 b
Kal-SP 5%	4.67 ± 4.42 a	32.00 ± 11.50 a	78.72 ± 24.14 a
Kal-SP 7%	1.72 ± 1.64 b	24.44 ± 13.80 b	78.11 ± 16.34 a
Control SC	0.61 ± 0.85 c	9.33 ± 10.20 d	22.00 ± 12.05 d
Kal-SC 1%	1.39 ± 1.29 b	15.11 ± 9.23 c	30.50 ± 12.23 c
Kal-SC 3%	2.11 ± 1.64 a	19.83 ± 9.57 b	32.44 ± 14.35 c
Kal-SC 5%	4.61 ± 5.04 a	28.83 ± 13.18 a	49.89 ± 21.12 b
Kal-SC 7%	3.44 ± 5.10 a	33.78 ± 20.38 a	67.89 ± 18.45 a

Means followed by distinct letters in the columns (for Kal-SP and Kal-SC individually) differ statistically (Scott and Knott test, *p* < 0.05).

**Table 2:** Mean value and standard deviation (±) of plant height (PH—cm), root length (RL—cm), number of branches (NB—n), number of nodes (NN—n), root fresh mass (RFW—g), shoot fresh mass (SFM—g), root dry mass (RDM—g), and shoot dry mass (SDM—g) of basil plants treated with foliar application of *Kappaphycus alvarezii* biostimulant (1%, 3%, 5%, and 7%) cultivated in the States of São Paulo (Kal-SP) and Santa Catarina (Kal-SC), Brazil.

Treatment	PH	RL	NB	NN	RFM	SFM	RDM	SDM
Control SP	77.33 ± 7.89 c	22.17 ± 4.02 a	2.56 ± 0.62 ^*ns^	12.61 ± 1.75 b	32.67 ± 9.68 b	297.72 ± 100.47 b	11.06 ± 4.21 b	52.83 ± 11.96 b
Kal-SP 1%	79.08 ± 5.59 c	17.56 ± 2.70 b	2.78 ± 1.00	13.78 ± 1.93 a	34.39 ± 9.52 b	345.67 ± 93.51 b	11.56 ± 4.08 b	51.67 ± 19.75 b
Kal-SP 3%	81.44 ± 10.66 c	17.92 ± 4.03 b	2.78 ± 0.88	14.00 ± 1.78 a	35.22 ± 10.51 b	386.72 ± 132.80 a	10.50 ± 3.29 b	48.50 ± 26.44 b
Kal-SP 5%	88.86 ± 4.00 a	20.67 ± 5.18 a	2.50 ± 0.62	13.06 ± 1.06 b	44.67 ± 13.79 a	450.11 ± 119.38 a	14.67 ± 6.36 a	82.83 ± 14.85 a
Kal-SP 7%	84.93 ± 4.34 b	24.25 ± 4.65 a	2.56 ± 0.62	13.06 ± 1.55 b	44.11 ± 8.09 a	444.61 ± 71.82 a	14.61 ± 2.87 a	83.83 ± 13.45 a
Control SC	73.09 ± 5.30 c	26.44 ± 7.18 a	2.83 ± 0.71 a	12.33 ± 2.38 b	41.33 ± 8.55 b	290.61 ± 98.74 b	14.28 ± 4.73 ^*ns^	42.67 ± 11.13 ^*ns^
Kal-SC 1%	79.71 ± 5.54 b	19.73 ± 3.80 b	2.83 ± 1.04 a	12.06 ± 1.80 b	35.61 ± 11.22 b	406.94 ± 117.64 a	11.11 ± 4.19	60.83 ± 17.15
Kal-SC 3%	85.76 ± 5.45 a	19.50 ± 5.09 b	2.28 ± 0.67 b	13.17 ± 1.42 a	42.11 ± 9.26 b	427.39 ± 103.76 a	13.89 ± 4.24	59.50 ± 19.16
Kal-SC 5%	86.21 ± 4.48 a	21.00 ± 4.54 b	2.39 ± 0.78 b	12.67 ± 1.50 b	40.67 ± 12.33 b	440.06 ± 96.86 a	13.06 ± 3.96	69.00 ± 21.53
Kal-SC 7%	83.57 ± 4.38 b	22.63 ± 5.37 a	2.78 ± 0.81 a	13.67 ± 1.50 a	48.61 ± 13.08 a	420.56 ± 84.83 a	16.44 ± 7.11	73.33 ± 20.52

Means followed by distinct letters in the columns (for Kal-SP and Kal-SC individually) differ statistically (Scott and Knott test, *p* < 0.05). ^*ns^—not significant.

**Table 3:** Mean value and standard deviation (±) of chlorophyll *a* (Chl *a*—mg/g), chlorophyll *b* (Chl *b*—mg/g), total chlorophyll (TChl—mg/g), total carotenoid (TCN—mg/g), total phenolic content (TPC—mg/g), total flavonoid content (TFC—mg/g), and DPPH antioxidant activity (%) of basil plants treated with foliar spraying of *Kappaphycus alvarezii* biostimulant (1%, 3%, 5%, and 7%) cultivated in the States of São Paulo (Kal-SP) and Santa Catarina (Kal-SC), Brazil.

Treatment	Chl *a*	Chl *b*	TChl	TCN	TPC	TFC	DPPH
Control SP	0.46 ± 0.08 ^*ns^	0.16 ± 0.03 ^*ns^	0.62 ± 0.12 ^*ns^	0.098 ± 0.017 ^*ns^	30.09 ± 6.78 a	0.70 ± 0.08 b	80.06 ± 0.57 ^*ns^
Kal-SP 1%	0.44 ± 0.05	0.16 ± 0.02	0.60 ± 0.07	0.094 ± 0.011	24.72 ± 9.09 b	0.90 ± 0.11 a	77.71 ± 2.76
Kal-SP 3%	0.49 ± 0.05	0.20 ± 0.10	0.68 ± 0.12	0.089 ± 0.028	27.13 ± 3.61 a	0.76 ± 0.12 b	79.00 ± 0.88
Kal-SP 5%	0.46 ± 0.03	0.16 ± 0.01	0.62 ± 0.04	0.097 ± 0.006	24.77 ± 2.92 b	0.68 ± 0.08 b	79.27 ± 0.63
Kal-SP 7%	0.46 ± 0.05	0.22 ± 0.13	0.64 ± 0.06	0.100 ± 0.006	23.80 ± 7.69 b	0.86 ± 0.15 a	78.21 ± 4.75
Control SC	0.40 ± 0.07 b	0.14 ± 0.02 c	0.54 ± 0.09 c	0.083 ± 0.013 b	19.43 ± 3.79 b	0.58 ± 0.10 ^*ns^	73.82 ± 6.14 b
Kal-SC 1%	0.43 ± 0.03 b	0.15 ± 0.01 c	0.58 ± 0.04 c	0.085 ± 0.007 b	22.30 ± 5.05 a	0.62 ± 0.10	73.79 ± 5.53 b
Kal-SC 3%	0.46 ± 0.07 a	0.16 ± 0.02 b	0.62 ± 0.09 b	0.095 ± 0.010 a	19.94 ± 5.81 b	0.62 ± 0.11	73.15 ± 6.26 b
Kal-SC 5%	0.51 ± 0.04 a	0.18 ± 0.02 a	0.68 ± 0.05 a	0.099 ± 0.005 a	22.21 ± 7.29 a	0.61 ± 0.16	73.29 ± 7.77 b
Kal-SC 7%	0.47 ± 0.02 a	0.16 ± 0.01 b	0.63 ± 0.03 b	0.096 ± 0.004 a	19.26 ± 5.20 b	0.56 ± 0.09	76.85 ± 1.67 a

Means followed by distinct letters in the columns (for Kal-SP and Kal-SC individually) differ statistically according to the Scott and Knott test (*p* < 0.05). ^*ns^—not significant.

**Table 4:** Mean value and standard deviation (±) of total soluble sugars (TSS—mg/g), total starch (TS—mg/g), total carbohydrate (TC—mg/g), total protein (TP—mg/g), and total amino acid (TAA—mg/g) of basil plants treated with foliar application of *Kappaphycus alvarezii* biostimulant (1%, 3%, 5%, and 7%) cultivated in the States of São Paulo (Kal-SP) and Santa Catarina (Kal-SC), Brazil.

Treatment	TSS	TS	TC	TP	TAA
Control SP	25.20 ± 4.20 a	5.74 ± 2.12 a	31.07 ± 1.61 ^*ns^	1.46 ± 0.21 a	6.93 ± 2.08 b
Kal-SP 1%	23.58 ± 8.38 b	5.83 ± 1.10 a	33.59 ± 0.93	0.97 ± 0.31 c	8.18 ± 2.48 a
Kal-SP 3%	19.22 ± 3.30 b	6.45 ± 4.17 a	35.16 ± 0.80	1.21 ± 0.22 b	8.73 ± 0.91 a
Kal-SP 5%	21.92 ± 7.82 b	3.87 ± 1.28 b	30.80 ± 0.94	1.25 ± 0.14 b	9.16 ± 1.91 a
Kal-SP 7%	28.42 ± 4.56 a	3.43 ± 0.86 b	33.66 ± 1.94	1.03 ± 0.19 c	9.69 ± 3.36 a
Control SC	25.97 ± 4.87 a	3.05 ± 1.61 a	29.06 ± 5.26 b	1.35 ± 0.10 a	4.84 ± 1.10 c
Kal-SC 1%	17.07 ± 2.93 b	2.03 ± 0.93 b	29.22 ± 2.99 b	0.90 ± 0.46 b	5.95 ± 1.42 b
Kal-SC 3%	22.01 ± 5.00 b	2.57 ± 0.80 b	33.13 ± 3.97 a	0.77 ± 0.38 c	7.60 ± 1.14 a
Kal-SC 5%	20.39 ± 8.75 b	2.35 ± 0.94 b	32.59 ± 3.97 a	0.70 ± 0.25 c	7.25 ± 1.31 a
Kal-SC 7%	25.61 ± 6.75 a	3.56 ± 1.94 a	33.00 ± 5.08 a	0.66 ± 0.39 c	7.41 ± 1.52 a

Means followed by distinct letters in the columns (for Kal-SP and Kal-SC individually) differ statistically (Scott & Knott test, *p* < 0.05). ^*ns^—not significant.

**Table 5:** Area (%) of metabolites identified by GC-MS in the basil essential oil fractions extracted from plants treated with foliar application of *Kappaphycus alvarezii* biostimulant (1%, 3%, 5%, and 7%) cultivated in São Paulo (Kal-SP) and Santa Catarina (Kal-SC), Brazil.

RT	Treatment	Control SP	Kal-SP 1%	Kal-SP 3%	Kal-SP 5%	Kal-SP 7%	Control SC	Kal-SC 1%	Kal-SC 3%	Kal-SC 5%	Kal-SC 7%
3.66–3.68	1,8-Cineole	2.19	1.09	1.30	1.02	1.39	2.43	1.10	2.54	1.63	1.73
4.31–4.33	β-Terpineol	1.38	0.56	0.58	0.45	0.59	0.66	0.52	0.89	0.56	0.68
6.90–7.01	Terpinen-4-ol	3.18	2.54	1.57	3.96	0.00	5.75	2.64	3.59	3.30	2.27
7.31–7.40	α-Terpineol	1.49	0.87	0.81	1.00	0.90	0.92	0.88	1.15	1.02	1.02
10.71–10.87	α-Pinene/β-Pinene	0.71	0.30	0.31	4.02	3.76	7.02	0.00	0.00	4.03	0.00
10.03–13.20	Eugenol	13.43	14.91	14.20	10.93	9.14	7.63	11.84	10.67	11.48	6.97
15.82–16.01	α-Farnesene	14.35	11.61	14.82	10.50	10.22	11.13	11.74	14.18	13.42	9.79
16.22–16.23	α-Humulene	0.00	0.00	0.00	0.00	0.25	0.00	0.00	0.31	0.30	0.00
17.13–17.19	Germacrene D	0.87	0.56	0.67	0.69	0.83	1.01	0.62	0.72	0.88	0.00
17.45–17.51	β-Farnesene	1.00	1.01	1.36	0.83	0.94	0.00	1.07	1.03	1.00	0.85
18.45–18.59	Cadinane-type sesquiterpene	5.74	7.12	7.91	4.96	6.01	4.92	5.35	5.92	5.69	5.11
18.70–18.77	Cadinane-type sesquiterpene	0.89	0.90	1.00	0.66	0.90	0.00	0.74	0.76	0.74	0.69
18.99–19.06	β-Caryophyllene	1.12	0.94	0.87	0.31	0.50	0.00	0.29	0.68	0.60	0.55
19.00–19.18	(Z,Z)-α-farnesene	0.00	0.00	0.36	0.00	0.36	0.00	0.00	0.31	0.32	0.51
20.58–20.67	Spathulenol	1.27	2.00	1.75	1.69	2.63	1.65	2.07	1.32	1.28	1.99
21.04–21.14	Guaiane-type sesquiterpene	0.48	1.19	0.00	1.10	1.50	1.11	1.57	0.71	0.72	0.87
21.99–22.12	Cadinane-type sesquiterpene	2.45	3.05	2.99	3.60	3.33	2.59	3.32	2.32	2.19	3.96
23.18–23.41	epi-Bicyclosesquiphellandrene	13.94	14.74	17.19	23.70	20.10	21.59	24.83	16.55	15.00	22.41
23.36–23.39	Cadinane-type sesquiterpene	0.00	0.00	0.41	0.00	0.39	0.00	0.58	0.39	0.37	0.60
23.40–23.54	Cadinol/Muurolol	0.85	0.74	0.98	1.41	1.07	1.02	1.24	0.83	0.83	1.34
30.45–30.53	Hexahydrofarnesyl acetone	3.47	2.79	3.54	4.13	1.13	1.61	1.90	1.78	2.64	3.91
38.61–38.70	Phytol	2.53	4.08	4.09	5.13	3.14	5.46	3.53	1.39	1.54	4.21
	Total area	71.33	71.00	76.70	80.09	69.07	76.50	75.83	68.04	69.54	69.46

Heatmap colors were calculated per column (variable) through the highest values (red), medium values (yellow), and the lowest values (green).

**Table 6:** Mean value and standard deviation (±) of area (%) of the major compounds found in the essential oil fraction extracted from basil leaves treated with foliar applications of *Kappaphycus alvarezii* biostimulants (1%, 3%, 5%, and 7%) and cultivated in São Paulo (SP) and Santa Catarina (SC), Brazil.

Treatments	Epi-Bicyclosesquiphellandrene	α-Farnesene	Eugenol
Control SP	13.94 ± 0.43 b	14.35 ± 2.41 a	13.43 ± 1.84 a
Kal-SP 1%	14.74 ± 3.70 b	11.61 ± 2.52 b	14.91 ± 0.84 a
Kal-SP 3%	17.19 ± 3.04 b	14.82 ± 0.72 a	14.20 ± 2.13 a
Kal-SP 5%	23.70 ± 2.58 a	10.50 ± 3.33 b	10.93 ± 1.77 b
Kal-SP 7%	20.10 ± 0.43 a	10.22 ± 0.62 b	9.14 ± 0.42 b
Control SC	21.59 ± 0.57 a	11.13 ± 0.16 b	7.63 ± 0.05 b
Kal-SC 1%	24.83 ± 1.18 a	11.74 ± 0.82 b	11.84 ± 1.50 a
Kal-SC 3%	16.55 ± 0.13 b	14.18 ± 0.36 a	10.67 ± 0.20 a
Kal-SC 5%	15.00 ± 0.95 b	13.42 ± 0.10 a	11.48 ± 0.06 a
Kal-SC 7%	22.41 ± 5.22 a	9.79 ± 0.62 b	6.97 ± 0.74 b

Means followed by distinct letters in the columns (for Kal-SP and Kal-SC individually) differ statistically according to the Scott & Knott test (*p* < 0.05).

## Data Availability

The data presented in this study are available upon request to the corresponding author due to privacy and the amount of data generated.

## Supplementary Materials

The following supporting information can be downloaded at: https://www.mdpi.com/article/10.3390/plants15111749/s1, Figure S1: Collection sites of the *Kappaphycus alvarezii* in Ubatuba, São Paulo (SP), and Florianópolis, Santa Catarina (SC); Figure S2: Rainfall precipitation (mm), relative humidity (%), and air temperature (°C) from 27 January 2025 to 17 April 2025, in the municipality of Florianópolis, southern Brazil; Figure S3: Representative gas chromatogram coupled with mass spectrometry (GC–MS) of basil leaf essential oils (treated with foliar application of *Kappaphycus alvarezii* biostimulant at 1%, 3%, 5%, and 7%) cultivated in the states of São Paulo (Kal–SP) and Santa Catarina (Kal–SC), Brazil; Figure S4: Principal component analysis (PC2 and PC3) of the metabolite profiles of essential oil extracted from basil leaves treated with foliar applications of *Kappaphycus alvarezii* biostimulants (1%, 3%, 5%, and 7%) and cultivated in São Paulo (SP) and Santa Catarina (SC); Table S1: Soil chemical and physical parameters, limit of detection (LD), and limit of quantification (LQ) prior to *Ocimum basilicum* cultivation. Table S2: Kaiser–Meyer–Olkin (KMO) index and Bartlett’s test of sphericity for each PCA performed in the study. Table S3: Alpha diversity of the rhizosphere of basil plants cultivated with a 7% biostimulant of *Kappaphycus alvarezii* from São Paulo (Kal-SP) and Santa Catarina (Kal-SC), Brazil; Table S4: Mean and standard deviation of the relative abundance (%) of phyla, classes, orders, and families in the rhizosphere of basil plants cultivated with a 7% biostimulant of *Kappaphycus alvarezii* from São Paulo (Kal-SP) and Santa Catarina (Kal-SC), Brazil.
