# Supplementary material for: Impact of Kappaphycus alvarezii Biostimulant on Growth, Biochemistry, Essential Oil, and Rhizosphere of Basil (Ocimum basilicum) Plants
Source: Plants (Basel). 2026 Jun 4;15(11):1749. doi: 10.3390/plants15111749 (PMC13259351; doi:10.3390/plants15111749)
Supplement: Supplementary file 1 [file plants-15-01749-s001.zip › plants-4359371-supplementary.pdf]

Supplementary material

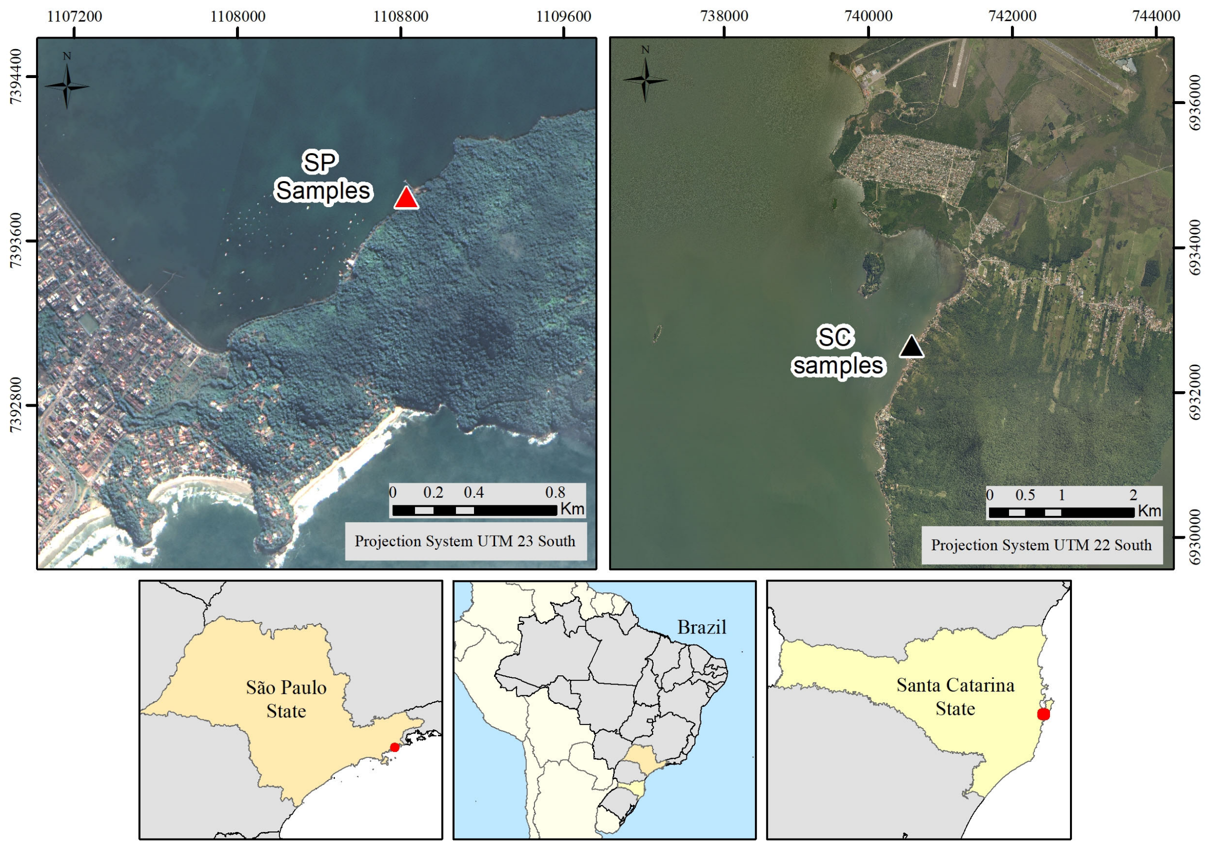

Figure S1. Collection sites of the *Kappaphycus alvarezii* in Ubatuba, São Paulo (SP), and Florianópolis, Santa Catarina (SC).

Source: Elaborated by the author (2025).

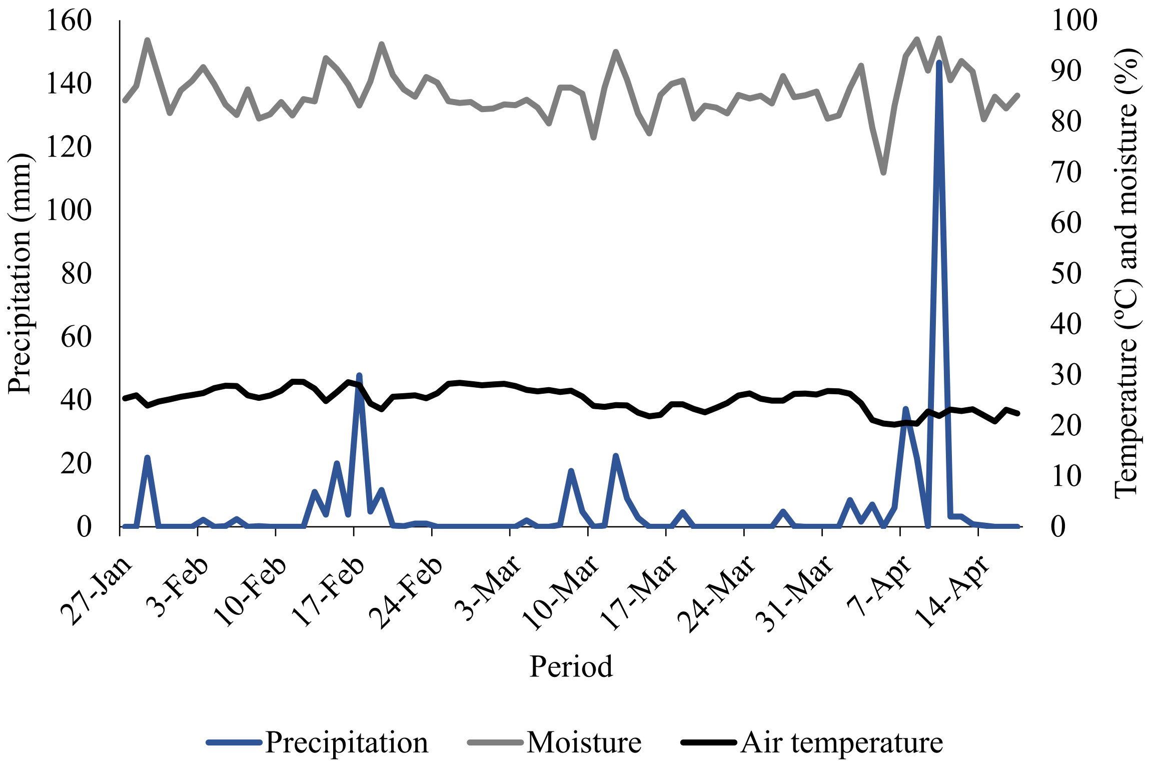

Figure S2. Precipitation (mm), relative humidity (%), and air temperature (°C) from January 27, 2025, to April 17, 2025, in the municipality of Florianópolis, southern Brazil.

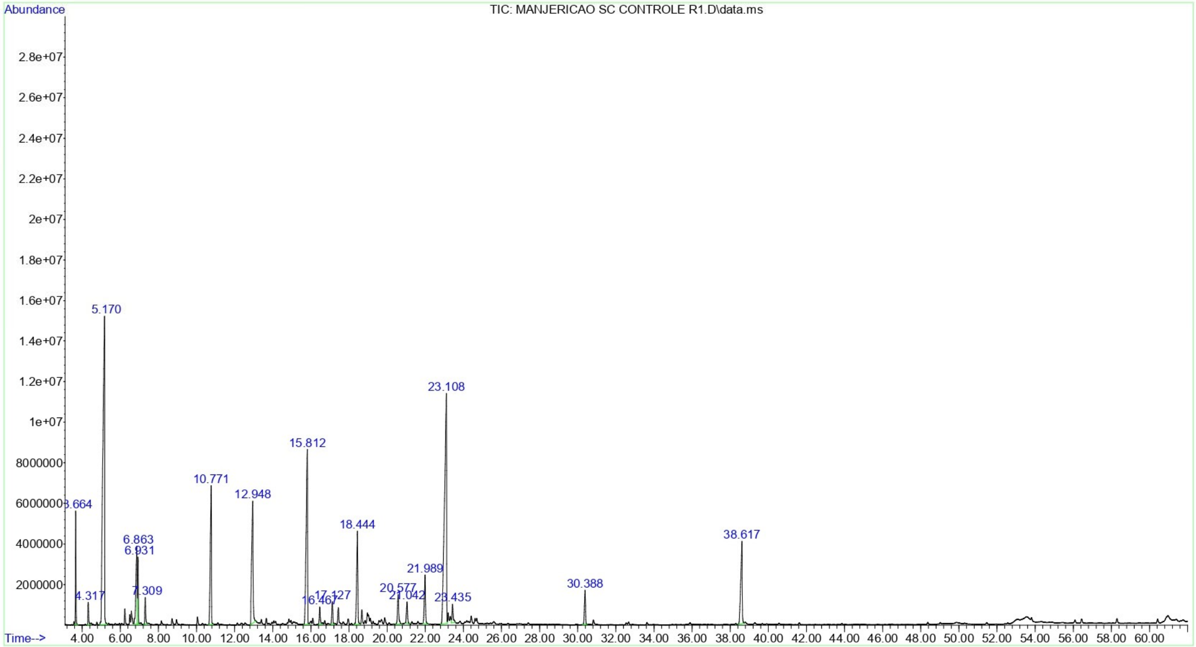

Figure S3. Representative gas chromatogram coupled with mass spectrometry (GC–MS) of basil leaf essential oils (treated with foliar application of Kappaphycus alvarezii biostimulant at 1%, 3%, 5%, and 7%) cultivated in the states of São Paulo (Kal–SP) and Santa Catarina (Kal–SC), Brazil.

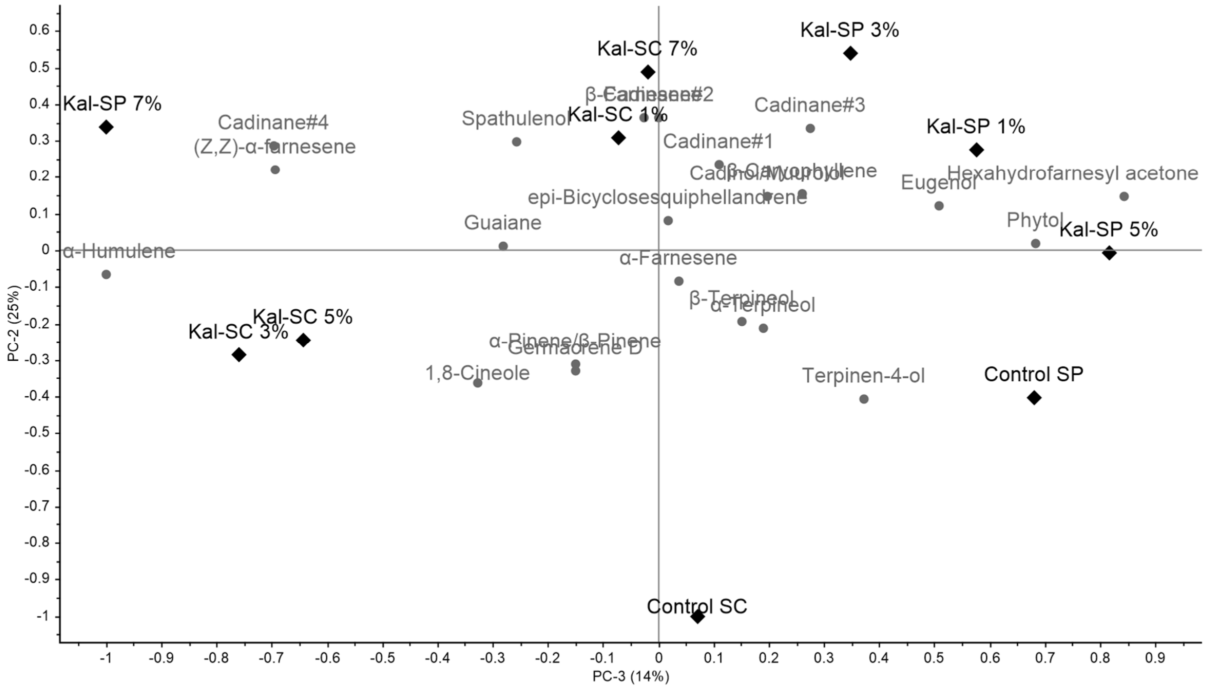

Figure S4. Principal component analysis (PC2 and PC3) based on the essential oil profiles extracted from basil leaves treated with foliar applications of *Kappaphycus alvarezii* biostimulants (1%, 3%, 5%, and 7%) and cultivated in São Paulo (SP) and Santa Catarina (SC).

Table S1. Soil chemical and physical parameters, limit of detection (LD), and limit of quantification (LQ) prior to *Ocimum basilicum* cultivation.

Parameters	Soil	LQ	LD
Clay (%)	40	3	-
pH (1:1)	5.2	-	-
SMP index	5.8	-	-
Phosphorus (mg/dm^3)	8.16	0.15	0.10
Potassium (mg/dm^3)	163.95	4.50	1.20
Organic matter (%)	3.15	0.53	0.12
Aluminum (cmolc/dm^3)	0.13	0.01	0.002
Calcium (cmolc/dm^3)	3.10	0.05	0.10
Magnesium (cmolc/dm^3)	1.77	0.01	0.002
Potential acidity (cmolc/dm^3)	18.31	-	-
Base saturation (%)	5.49	-	-
Iron (mg/dm^3)	49.09	-	-
Copper (mg/dm^3)	190.80	1.19	-
Zinc (mg/dm^3)	32.11	0.25	-
Boron (mg/dm^3)	3.37	0.08	-
Sulfur (mg/dm^3)	4.31	0.63	-
Phosphorus (mg/dm^3)	0.38	0.22	-
Potassium (mg/dm^3)	8.50	4.54	-

LQ – Limit of quantification; LD - Limit of detection.

Table S2. Kaiser–Meyer–Olkin (KMO) index and Bartlett's test of sphericity for each PCA analysis performed in the study.

PCA Analysis	Variables Included	KMO	Bartlett's χ^2	df	p-value
Figure 1	Morphological parameters	0.32	112.84	36	<0.001
Figure 2	Biochemical parameters	0.50	491.64	66	<0.001
Figure 4	Essential oil	0.50	394.13	231	<0.001
Figure 6	Rhizosphere microbiome	0.50	Not valid*	153	-

Table S3. Alpha diversity of the rhizosphere of basil plants cultivated with a 7% biostimulant of *Kappaphycus alvarezii* from São Paulo (Kal-SP) and Santa Catarina (Kal-SC), Brazil.

	Control SP	Kal-SP 7%	Control SC	Kal-SC 7%
Taxa_S	1188	1237	1203	875
Individuals	67107	65126	62904	48202
Dominance_D	0.0028	0.0034	0.0029	0.0037
Simpson_1-D	0.9972	0.9967	0.9971	0.9963
Shannon_H	6.304	6.160	6.261	6.030
Evenness_e^H/S	0.467	0.409	0.442	0.502
Brillouin	6250	6106	4124	5976
Menhinick	4598	4813	4783	3950
Margalef	107	111	109	81
Equitability_J	0.891	0.869	0.883	0.894
Fisher_alpha	205	218	211	152
Berger-Parker	0.0093	0.0130	0.0098	0.0130
Chao-1	1188	1237	1203	875
iChao-1	1188	1237	1203	875
ACE	1188	1239	1204	876
Squares	1188	1237	1203	875

Table S4. Mean and standard deviation of the relative abundance (%) of phyla, classes, orders, and families in the rhizosphere of basil plants cultivated with a 7% biostimulant of *Kappaphycus alvarezii* from São Paulo (Kal-SP) and Santa Catarina (Kal-SC), Brazil.

Taxonomy	Dominant bacteria	Control SP	Kal-SP 7%	Control SC	Kal-SC 7%
Phylum	Proteobacteria	40.61 ± 5.41 *ns	44.61 ± 1.27	43.57 ± 0.97 *ns	39.11 ± 5.89
	Actinobacteriota	17.32 ± 2.44 *ns	14.28 ± 1.67	14.18 ± 1.09 *ns	15.03 ± 0.93
	Acidobacteriota	11.63 ± 1.07 a	9.62 ± 0.80 b	9.46 ± 0.99 *ns	11.53 ± 4.57
	Chloroflexi	5.80 ± 1.21 *ns	6.76 ± 1.34	5.93 ± 0.58 *ns	19.10 ± 3.33
	Bacteroidota	5.81 ± 0.93 *ns	7.12 ± 0.98	7.02 ± 1.27 *ns	5.43 ± 1.23
	Firmicutes	5.05 ± 2.45 *ns	5.85 ± 0.10	5.39 ± 0.61 *ns	6.95 ± 2.94
	Myxococcota	4.31 ± 1.07 *ns	2.89 ± 0.28	4.87 ± 1.37 *ns	3.89 ± 1.99
	Gemmatimonadota	1.63 ± 0.43 *ns	2.04 ± 0.38	2.28 ± 0.50 *ns	1.22 ± 0.85
	Patescibacteria	1.72 ± 0.63 *ns	2.01 ± 0.14	1.80 ± 0.29 *ns	1.54 ± 0.36
	Verrucomicrobiota	2.04 ± 0.29 *ns	1.68 ± 0.71	2.03 ± 0.26 *ns	2.39 ± 0.19
	Planctomycetota	1.04 ± 0.22 *ns	0.63 ± 0.25	0.86 ± 0.21 *ns	0.97 ± 0.20

Overall average dissimilarity		7.85		8.38	
Class	Alphaproteobacteria	28.88 ± 6.21 *ns	31.84 ± 1.38	31.50 ± 1.38 *ns	28.66 ± 2.68
	Gammaproteobacteria	11.74 ± 1.00 *ns	12.79 ± 1.55	12.08 ± 0.43 *ns	10.96 ± 3.71
	Actinobacteria	13.52 ± 2.06 *ns	11.21 ± 1.57	11.26 ± 1.17 *ns	10.86 ± 0.35
	Acidobacteriae	9.41 ± 0.13 a	7.98 ± 0.78 b	7.84 ± 0.68 *ns	9.68 ± 5.16
	Bacteroidia	5.70 ± 0.87 *ns	7.09 ± 0.98	7.00 ± 1.26 *ns	5.63 ± 1.47
	Bacilli	3.83 ± 1.86 *ns	5.15 ± 0.20	4.50 ± 0.74 *ns	6.19 ± 2.26
	Ktedonobacteria	3.42 ± 0.92 *ns	3.47 ± 0.60	3.23 ± 0.22 *ns	5.80 ± 4.32
	Gemmatimonadetes	1.54 ± 0.47 *ns	1.93 ± 0.37	2.12 ± 0.42 *ns	1.22 ± 0.83
	Polyangia	2.92 ± 0.48 a	1.91 ± 0.18 b	3.03 ± 0.84 *ns	2.60 ± 1.30
	Saccharimonadia	1.69 ± 0.61 *ns	1.96 ± 0.12	1.78 ± 0.29 *ns	1.57 ± 0.46
	Thermoleophilia	2.61 ± 0.56 *ns	2.25 ± 0.48	2.15 ± 0.27 *ns	3.24 ± 1.50
	Verrucomicrobiae	1.96 ± 0.35 *ns	1.65 ± 0.68	1.98 ± 0.26 b	2.43 ± 0.14 a
	Chloroflexia	0.67 ± 0.08 b	1.18 ± 0.23 a	0.89 ± 0.42 *ns	0.88 ± 0.46
	Myxococcia	1.31 ± 0.73 *ns	0.88 ± 0.09	1.75 ± 0.65 *ns	1.16 ± 0.53
	Vicinamibacteria	1.35 ± 0.94 *ns	1.09 ± 0.21	1.21 ± 0.21 *ns	1.62 ± 1.48
	Acidimicrobiia	1.13 ± 0.34 *ns	0.81 ± 0.33	0.71 ± 0.38 b	1.50 ± 0.25 a
Overall average dissimilarity		9.30		9.29	
Order	Rhizobiales	15.60 ± 3.57 *ns	15.46 ± 2.51	15.21 ± 1.28 *ns	14.66 ± 2.40
	Burkholderiales	5.02 ± 1.09 *ns	4.68 ± 0.63	5.19 ± 1.31 *ns	4.32 ± 2.31
	Acidobacteriales	4.89 ± 0.71 a	3.62 ± 0.41 b	4.26 ± 0.77 *ns	5.35 ± 2.11
	Chitinophagales	4.16 ± 0.78 *ns	4.74 ± 1.12	4.22 ± 1.15 *ns	3.24 ± 0.90
	Micropepsales	2.68 ± 0.87 b	3.95 ± 0.50 a	3.98 ± 0.36 a	2.06 ± 0.38 b
	Xanthomonadales	3.06 ± 0.87 *ns	3.59 ± 1.15	3.70 ± 0.78 a	2.44 ± 0.33 b
	Sphingomonadales	2.41 ± 0.84 *ns	3.08 ± 1.38	2.30 ± 0.80 *ns	1.69 ± 0.74
	Ktedonobacterales	3.12 ± 0.75 *ns	3.21 ± 0.64	3.02 ± 0.22 *ns	5.34 ± 4.51
	Bacillales	1.97 ± 0.63 *ns	2.99 ± 0.46	2.36 ± 0.14 b	4.17 ± 1.78 a
	Micromonosporales	1.70 ± 0.64 *ns	1.48 ± 0.39	1.68 ± 0.78 *ns	0.92 ± 0.41
	Caulobacterales	1.40 ± 0.28 *ns	1.25 ± 0.20	1.73 ± 0.51 a	1.05 ± 0.18 b
	Micrococcales	2.71 ± 0.11 *ns	1.73 ± 0.11	1.93 ± 0.45 *ns	1.59 ± 0.88
	Gemmatimonadales	1.56 ± 0.48 *ns	1.94 ± 0.37	2.14 ± 0.43 *ns	1.19 ± 0.85
	Streptosporangiales	2.09 ± 0.46 *ns	2.83 ± 0.47	2.25 ± 0.43 *ns	1.60 ± 0.81
	Elsterales	1.59 ± 0.74 *ns	1.57 ± 0.36	1.77 ± 0.33 b	3.59 ± 2.07 a
	Saccharimonadales	1.70 ± 0.62 *ns	1.97 ± 0.12	1.79 ± 0.30 *ns	1.51 ± 0.35
	Streptomycetales	2.29 ± 0.44 *ns	1.99 ± 0.20	1.75 ± 0.24 *ns	1.80 ± 1.17
	Bryobacterales	2.15 ± 0.68 *ns	2.46 ± 0.24	2.01 ± 0.35 *ns	1.73 ± 0.81
	Cytophagales	0.80 ± 0.07 b	1.05 ± 0.20 a	1.62 ± 0.22 *ns	1.28 ± 0.75
	Frankiales	1.75 ± 0.21 a	0.89 ± 0.39 b	1.18 ± 0.32 *ns	1.96 ± 1.21
	Gaiellales	1.68 ± 0.25 *ns	1.57 ± 0.49	1.44 ± 0.10 *ns	1.72 ± 0.74
	Solibacterales	1.68 ± 0.67 *ns	1.51 ± 0.52	1.06 ± 0.27 *ns	1.41 ± 1.32
	Myxococcales	1.32 ±0.73 *ns	0.88 ± 0.09	1.76 ± 0.66 *ns	1.12 ± 0.52
	Polyangiales	1.29 ± 0.08 *ns	1.17 ± 0.31	1.62 ± 0.36 *ns	1.73 ± 1.21
	Vicinamibacterales	1.34 ± 0.98 *ns	1.10 ± 0.21	1.21 ± 0.23 *ns	1.57 ± 1.48
	Acetobacterales	1.95 ± 0.70 *ns	1.65 ± 0.08	1.77 ± 0.60 *ns	1.63 ± 0.25
	Paenibacillales	0.92 ± 0.55 *ns	1.00 ± 0.37	1.22 ± 0.53 *ns	0.96 ± 0.84
	Sphingobacteriales	0.69 ± 0.45 *ns	1.24 ± 0.27	1.05 ± 0.29 *ns	0.82 ± 0.49

	Reyranellales	1.02 ± 0.59 *ns	1.14 ± 0.07	1.08 ± 0.10 *ns	0.88 ± 0.22
Overall average dissimilarity		15.67		12.82	
Family	Xanthobacteraceae	6.14 ± 2.53 *ns	5.76 ± 2.05	5.50 ± 1.27 *ns	7.25 ± 1.19
	Chitinophagaceae	4.12 ± 0.78 *ns	4.65 ± 1.10	4.19 ± 1.21 *ns	3.27 ± 0.94
	Micropepsaceae	2.71 ± 0.89 *ns	3.99 ± 0.52	4.04 ± 0.37 a	2.08 ± 0.39 b
	Acidobacteriaceae	2.74 ± 0.72 *ns	2.44 ± 0.27	2.73 ± 0.65 *ns	2.27 ± 0.87
	Sphingomonadaceae	2.44 ± 0.85 *ns	3.11 ± 1.37	2.33 ± 0.82 *ns	1.71 ± 0.75
	Rhodanobacteraceae	2.87 ± 0.92 *ns	3.05 ± 1.02	3.14 ± 0.66 *ns	2.19 ± 0.46
	Rhizobiaceae	2.22 ± 0.62 *ns	1.88 ± 0.53	2.42 ± 0.46 *ns	1.54 ± 0.96
	Bacillaceae	1.92 ± 0.65 *ns	2.91 ± 0.54	2.34 ± 0.19 b	3.88 ± 1.21 a
	Micromonosporaceae	1.72 ± 0.65 *ns	1.50 ± 0.39	1.71 ± 0.79 *ns	0.93 ± 0.41
	Ktedonobacteraceae	2.38 ± 0.55 *ns	2.76 ± 0.64	2.46 ± 0.29 *ns	3.88 ± 3.17
	Comamonadaceae	0.79 ± 0.51 *ns	0.77 ± 0.27	1.34 ± 0.90 *ns	0.53 ± 0.38
	Gemmatimonadaceae	1.57 ± 0.49 *ns	1.96 ± 0.38	2.17 ± 0.43 *ns	1.21 ± 0.86
	Caulobacteraceae	1.16 ± 0.19 *ns	0.97 ± 0.13	1.51 ± 0.50 a	0.91 ± 0.27 b
	Streptomycetaceae	2.31 ± 0.44 *ns	2.02 ± 0.21	1.78 ± 0.25 *ns	1.82 ± 1.18
	Bryobacteraceae	2.18 ± 0.69 *ns	2.49 ± 0.23	2.04 ± 0.35 *ns	1.75 ± 0.81
	Beijerinckiaceae	1.70 ± 0.35 *ns	1.65 ± 0.58	1.88 ± 0.90 *ns	1.53 ±1.09
	Microscillaceae	0.62 ± 0.14 b	0.95 ± 0.17 a	1.45 ± 0.22 *ns	0.94 ± 0.50
	Hyphomicrobiaceae	1.86 ± 0.53 *ns	2.02 ± 0.68	1.96 ± 0.49 *ns	1.65 ± 0.19
	Streptosporangiaceae	1.24 ± 0.66 *ns	1.42 ± 0.12	1.39 ± 0.21 *ns	1.07 ± 0.51
	Solibacteraceae	1.70 ± 0.68 *ns	1.52 ± 0.52	1.08 ± 0.28 *ns	1.42 ± 1.32
	Acetobacteraceae	1.98 ± 0.71 *ns	1.66 ± 0.08	1.80 ± 0.60 *ns	1.65 ± 0.26
	Intrasporangiaceae	1.38 ± 0.25 *ns	1.20 ± 0.09	1.02 ± 0.42 *ns	1.03 ± 0.60
Overall average dissimilarity		20.8		15.6	

*ns – not significant according to the Kruskal-Wallis test ($p < 0.05$). Different letters on the same line indicate statistically significant differences as determined by the Kruskal-Wallis test ($p < 0.05$) when comparing each state individually (Control SP vs. Kal-SP 7%; Control SC vs. Kal-SC 7%).
